# A clinical, aetiological, and public health perspective on central nervous system infections in Bolivia, 2017–2018

**DOI:** 10.1038/s41598-021-02592-6

**Published:** 2021-12-01

**Authors:** Paola Mariela Saba Villarroel, María del Rosario Castro Soto, Oriana Melendres Flores, Alejandro Peralta Landívar, María E. Calderón, Roxana Loayza, José Boucraut, Laurence Thirion, Audrey Dubot-Pérès, Laetitia Ninove, Xavier de Lamballerie

**Affiliations:** 1 Unité des Virus Émergents (UVE: Aix-Marseille Univ.-IRD 190-INSERM 1207-IHU Méditerranée Infection), 13005 Marseille, France; 2 Molecular Biology Unit, Centro Nacional de Enfermedades Tropicales (CENETROP), Santa Cruz de la Sierra, Bolivia; 3 Infectology Department, Viedma Hospital, Cochabamba, Bolivia; 4 Neurology Department, Dr. Mario Ortíz Suárez Hospital, Santa Cruz de la Sierra, Bolivia; 5 Neurology Department, Japonés Hospital, Santa Cruz de la Sierra, Bolivia; 6 Infectology Department, Manuel Ascencio Villarroel Hospital, Cochabamba, Bolivia; 7 Immunology Laboratory, Conception Hospital, 13005 Marseille, France; 8 Timone Neuroscience Institute, Aix-Marseille University, 13005 Marseille, France

**Keywords:** Infectious-disease diagnostics, Bacterial infection, Fungal infection, HIV infections, Meningitis, Parasitic infection, Tuberculosis, Viral infection

## Abstract

Central nervous system (CNS) infections are important causes of morbidity and mortality worldwide. In Bolivia, aetiologies, case fatality, and determinants of outcome are poorly characterised. We attempted to investigate such parameters to guide diagnosis, treatment, prevention, and health policy. From Nov-2017 to Oct-2018, we prospectively enrolled 257 inpatients (20.2% HIV-positive patients) of all ages from healthcare centers of Cochabamba and Santa Cruz, Bolivia with a suspected CNS infection and a lumbar puncture performed. Biological diagnosis included classical microbiology, molecular, serological and immunohistochemical tests. An infectious aetiology was confirmed in 128/257 (49.8%) inpatients, including, notably among confirmed single and co-infections, *Cryptococcus* spp. (41.7%) and *Mycobacterium tuberculosis* (27.8%) in HIV-positive patients, and *Mycobacterium tuberculosis* (26.1%) and *Streptococcus pneumoniae* (18.5%) in HIV-negative patients. The total mortality rate was high (94/223, 42.1%), including six rabies cases. In multivariate logistic regression analysis, mortality was associated with thrombocytopenia (Odds ratio (OR) 5.40, 95%-CI 2.40–11.83) and hydrocephalus (OR 4.07, 95%-CI 1.35–12.23). The proportion of untreated HIV patients, late presentations of neurotuberculosis, the rate of pneumococcal cases, and rabies patients who did not benefit from a post-exposure prophylaxis, suggest that decreasing the burden of CNS infections requires reinforcing health policy regarding tuberculosis, rabies, *S. pneumoniae* vaccination, and HIV-infections.

## Introduction

A wide range of bacteria, viruses, fungi, and parasites can cause central nervous system (CNS) infections, which are important causes of mortality and long-term disability worldwide. The epidemiology and outcome of CNS infections depend on demographic factors, geographic region, immune status (including vaccination), and comorbidities^1^. Autoimmune diseases can also be the cause of encephalitis, which are indistinguishable from infectious aetiologies^2^. In previous large clinical studies, although extensive diagnostic testing was performed, aetiologies remained undiagnosed approximately in half of the patients^3^.

In developing countries, CNS infections represent a major challenge due to limited biological diagnostic methods (e.g. molecular assays), medical imaging, and healthcare access, which may lead to delay intervention and treatment.

In 2018, approximately one-third of the Bolivian population lived in poverty^4^, the health system was accessible to those covered by the social security (mostly salaried employees), and the public hospitals provided some free medical services for specific groups (e.g. elderly, children < 5 years old (yo), HIV-positive patients) not insured under any social security regimen^5^.

Little is known about the incidence and aetiologies of CNS infections in Bolivia. Previous limited reports point to the importance of Rabies^6^, a zoonotic vaccine-preventable viral disease, that remains endemic in Bolivia^7^ (743 suspected cases of rabies in dogs, 2018^8^); the importance of neurotuberculosis and neurocysticercosis in adults and children^9–11^ (incidence rate of all types of tuberculosis in Bolivia: 108 (71–154) per 100,000 inhabitants, mortality rate 11 (7.8–14) per 100,000 inhabitants in 2018^12^); and other studies also indicate that *Haemophilus influenzae, Streptococcus pneumoniae,* and *Neisseria meningitidis* are frequently reported as paediatric aetiologies^13–15^. Vaccination programs for *Haemophilus influenzae* and *Streptococcus pneumoniae* (PCV13) were introduced in 2000^16^ and 2014^17^, respectively.

In Bolivia, the estimated prevalence of Human Immunodeficiency viruses (HIV) is low (0.3% of the population aged between 15–49 years old^18^). However, Bolivia has one of the highest HIV-mortality rates in South America^19^. In acquired immune deficiency syndrome (AIDS) patients, *Cryptococcus* spp.^20^, *Trypanosoma cruzi*^21^¸ and *Toxoplasma gondii*^22^ infections have been reported. No consolidated data is available regarding to neuro-arboviral, enterovirus, herpes infections or autoimmune encephalitis.

Here, we conducted the first prospective study of CNS infections in Bolivia. We enrolled inpatients from the departments of Santa Cruz and Cochabamba and identified the most frequent aetiologies, which are discussed in relation with clinical, laboratory, and outcome parameters. Our results may allow guiding the public health strategy for management of CNS infections in Bolivia in the fields of diagnosis, treatment, and prevention.

## Methods

### Study sites and population

Between Nov-2017 and Oct-2018, we conducted a prospective study in Cochabamba (Andean valley, 2558 m above sea level (MASL). Population, 1,971,523) and Santa Cruz (hot and humid climate, 416 MASL. Population, 3,224,662), Bolivia. Both regions represented approximately half of the total Bolivian population^23^. The poverty rate was 32.9% in Cochabamba and 26.1% in Santa Cruz in 2018^4^.

We included mainly healthcare centers with the highest expertise level (public or private tertiary-level hospitals), that are neurological infections referral centers (Supplementary Table S1). The population studied mostly belonged to disadvantaged social groups (patients recruited from public hospitals: 85.6% in Cochabamba and 97.3% in Santa Cruz).

We enrolled inpatients of all ages suspected of CNS infection for whom lumbar puncture was performed at treating physician’s discretion. All identified patients who satisfied the eligibility criteria and signed the informed consent were enrolled. Patients’ demographics, medical history, clinical manifestations, examination findings, and treatment were collected from patients’ charts by the principal investigator and anonymized on standardized forms. Clinical outcome (mortality, sequelae, full recovery) was recorded at discharge and by contacting the relatives three, six, and for some patients 12 months after discharge, based on the Liverpool Outcome Score and adapted for children^24^.

### Ethical approval

Written informed consent was obtained from patients, close relatives, or parent/legal guardian in patients under the age of 18 years, prior to enrolment. Ethics committee of the Institut de Recherche pour le Développment (IRD), France, approved this study.

### Clinical case definition

We used for case definition and analysis the World Health Organization (WHO) criteria for encephalitis, meningitis, and meningoencephalitis as modified by Dubot-Pérès et al.^3^. Any patient with history of fever or axillary temperature > 37.5 °C and altered mental status, neck stiffness, seizures, or any combination of these features fulfilled the CNS infection criteria. Of note, the case definition for encephalitis met the criteria of the International Encephalitis Consortium^25^ with some limitations: results of electroencephalography and neuroimaging were not used due to limited access to these techniques.

### Neuroimaging

Medical imaging was proposed by physicians when needed. In public hospitals, computed tomography (CT) scan was free of charge for adults > 60 yo and children < 5 yo (for HIV-positive patients, depended on local social programs). In other cases, it was generally at family’s expenses.

### Laboratory assays

We aimed to collect six mL for adults (≥ 15 yo) and two mL for children (< 15 yo) of cerebrospinal fluid (CSF), two mL of serum, two mL of whole blood, and a nasopharyngeal swab (Sigma Virocult, Missouri, USA). Specimens were sent in cold chain (4 °C) to the National Center for Tropical Diseases (CENETROP), Santa Cruz, within 36 h to be aliquoted, immediately tested and stored at -80 °C for further molecular and serological analyses.

Blood count, biochemical tests, HIV tests, and CSF analysis including Gram, Ziehl–Neelsen stains, and microbiological culture, were performed at the hospital laboratories when indicated by the physician, and results were recorded.

CSF samples were prospectively tested for a first-line aetiological diagnostic panel of *Cryptococcus* spp. (Cryptococcal Antigen Latex System, Meridian Bioscience, Cincinnati, USA) and 13 or 18 common or treatable pathogens for HIV-negative or HIV-positive patients, respectively, using well-established non-commercial real-time PCR tests (Supplementary Table S2 and S3). Results were immediately made available to the treating physician.

Second-line diagnosis included Xpert MTB/RIF and Xpert MTB/RIF Ultra (Cepheid, California, USA) for tuberculosis in CSF; non-commercial real-time PCR or commercial serological assays for other pathogens in CSF or blood; commercial multiplex real-time PCR for respiratory pathogens (Fast-Track diagnostics, Luxemburg) in nasopharyngeal swabs; indirect immunohistochemistry and immunofluorescence tests (Euroimmun, Lübeck, Germany) in CSF and serum for autoimmune encephalitis. Tests were performed retrospectively except in case of specific clinical suspicion (*e.g.* rabies) (Supplementary Table S2 and S4).

For PCR assays, nucleic acids were extracted from 200 µl of samples (QIAamp MinElute Virus Spin or DNA Mini kit, Qiagen, Hilden, Germany), with a final elution volume of 150 μl. Bacteriophages MS2 and T4 were used as internal control^26^. Primers and probes were in a lyophilized format^27^ (Supplementary Table S3). Reactions were performed using Invitrogen SuperScript III reverse transcriptase (ThermoFisher Scientific, Massachusetts, USA) and a CFX96 thermal cycler (Bio-Rad, California, USA). All methods were performed in accordance with the relevant guidelines and regulations.

### Diagnostic interpretation

Based on laboratory results, cases were categorized as: infectious aetiologies ('confirmed', 'probable', or 'possible', see criteria in the supplementary table S5), non-infectious aetiologies (including confirmed autoimmune encephalitis), and unknown aetiologies. In brief, confirmed infectious cases mostly relied on direct detection of pathogens by polymerase chain reaction (PCR) in CSF, probable and possible infectious cases on pathogen detection in a specimen other than CSF or on serological results. Patients with a diagnosis other than CNS infection at discharge were classified as non-infectious cases (confirmed anti-NMDA receptor encephalitis was based on the presence of autoantibodies against neuronal surface proteins in serum and CSF by immunohistochemistry and immunofluorescence).

### Data analysis

Statistical analyses were performed according to sex, age-group (children < 15 yo, adults ≥ 15 yo), HIV status, study site, microorganism class, aetiologies, clinical features, blood and CSF parameters, clinical syndromes (meningitis, encephalitis, meningoencephalitis), and outcome, using IBM-SPSS v24.0.0.0 software. We compared variables using the Pearson chi-square or Fisher exact-test, and *t*-test or ANOVA for continuous variables. Univariate analysis was performed using demographic, aetiological, clinical, laboratory, and outcome parameters. Variables with p-values < 0.05 were included in multivariate logistic regression analysis of outcome parameters. Odds ratios were estimated with 95% confidence intervals (CI).

## Results

### Patients’ characteristics

Two hundred fifty-seven inpatients with suspected CNS infection were enrolled during the one-year study period (Table 1). In brief, 175/257 (68.1%) were adults (interquartile range (IQR): 10–51); 146/257 (56.8%) were recruited from Cochabamba; sex ratio was 1.5 (M/F); 52/257 (20.2%) were HIV-positive (IQR: 29–46, 42/52 (80.8%) recruited in Cochabamba, sex ratio 3.3 (M/F)). CD4 count was available for 30/52 (57.7%) HIV-positive patients, among whom CD4 count was < 200/µL for 26/30 (86.7%). At admission, median time from illness onset was seven days. Importantly, 20/257 (7.8%) patients reported domestic animal bite, of whom only 7/20 (35.0%) had received postexposure rabies prophylaxis.

**Table 1:** Characteristics of patients with suspected central nervous system infection by confirmed infectious aetiology and unknown aetiologies. Bolivia, 2017–2018. Data are presented as mean (range and interquartile range), or number (%).

	All patients	Confirmed infectious aetiology	Unknown aetiology	p-value infectious/unknown
	n = 257	n = 128	n = 46	
	no. (%)	no. (%)	no. (%)	
**Demographics**				
Male	156 (60.7)	87 (67.9)	27 (58.7)	0.256
Female	101 (39.3)	41 (32.0)	19 (41.3)	
Median age (range; interquartile range (IQR))	29 (0–88; 10–51)	29 (0–83; 12–53)	21 (0–88; 10–45)	0.135
Children (< 15 years old)	82 (31.9)	38 (29.7)	17 (36.9)	0.363
Adults (≥ 15 years old)	175 (68.1)	90 (70.3)	29 (63.0)	
Recruited from Cochabamba	146 (56.8)	78 (60.9)	19 (41.3)	0.378
Recruited from Santa Cruz	111 (43.2)	50 (39.1)	27 (58.7)	
**History**				
Human immunodeficiency virus (HIV)	52 (20.2)	36 (69.2)	6 (13.0)	**0.040**
Diabetes	21 (8.2)	10 (7.8)	2 (4.3)	0.426
Pulmonary tuberculosis (patient or family member)	29 (11.3)	19 (14.8)	4 (8.7)	0.290
Domestic animal bite or scratch	20 (7.8)	11 (8.6)	6 (13.0)	0.383
Median day of illness at admission (range; IQR range)	7 (0–210; 2–14)	7 (0–210; 2–15)	7 (0–90; 2.5–14)	0.419
Median day between admission & lumbar puncture (range; IQR range)	2 (0–61; 1–4)	1 (0–94; 1–4)	2 (0–23; 1–4)	0.181
Neuroimaging	171 (66.5)	84 (65.6)	35 (76.1)	0.190
**Clinical features**				
Fever > 37.5 °C	223 (86.8)	117 (91.4)	42 (91.3)	0.983
Glasgow Coma Scale < 12	85/227 (37.4)	48/112 (42.8)	12/42 (28.6)	0.105
Headache > 2.5 years of age	148/220 (67.3)	76/114 (66.7)	32 (69.6)	0.723
Nausea and/or vomiting	153 (59.5)	78 (60.9)	30 (65.2)	0.607
Neck stiffness or bulging fontanelle	113 (43.9)	78 (60.9)	24 (52.2)	0.355
Seizures	109 (42.4)	46 (35.9)	22 (47.8)	0.156
**Clinical syndromes**				
CNS infection criteria	208 (80.9)	112 (87.5)	41 (89.1)	0.770
Meningitis	53/208 (25.5)	38/112 (33.9)	7/41 (17.1)	**0.042**
Encephalitis	41/208 (19.7)	8/112 (7.1)	11/41 (26.8)	**0.001**
Meningoencephalitis	114/208 (54.8)	66/112 (58.9)	23/41 (56.1)	0.753
**Blood abnormalities**				
Hemoglobin < 12 g/dL	142 (55.2)	72 (56.2)	22 (47.8)	0.325
Leukocytosis > 10,000 cell/mm^3^	121 (47.1)	59 (46.1)	30 (65.2)	**0.026**
Neutrophilia > 65%	209 (81.3)	110 (85.9)	40 (86.9)	0.863
Postprandial hyperglycaemia ≥ 130 mg/dL	48/222 (21.6)	46/111 (41.4)	15/42 (35.7)	0.518
Thrombocytopenia < 150,000 cell/mm^3^	47/254 (18.5)	22/128 (17.2)	6/45 (13.3)	0.545
**Cerebrospinal fluid (CSF) abnormalities**				
Abnormal CSF	179 (69.6)	117 (91.3)	21 (45.7)	** < 0.001**
Hypoglycorrhachia < 40 mg/dL	151 (58.6)	106 (82.8)	20 (43.5)	** < 0.001**
Elevated proteins > 50 mg/dL	136/253 (53.8)	94/124 (75.8)	19 (41.3)	** < 0.001**
Elevated CSF leucocyte count > 10 cell/mm^3^	129 (50.2)	95 (74.2)	16 (34.8)	**0.041**
**Clinical outcome**				
Mortality	94/223 (42.1)	52/110 (47.3)	12/39 (30.8)	0.073
Full recovery	63/223 (28.3)	20/110 (18.2)	22/39 (56.4)	** < 0.001**
Sequelae in survivors	66/129 (51.2)	38/58 (65.5)	5/27 (18.5)	** < 0.001**

Significant values are in [bold]

Patients’ immunization record cards were available for review in 40/53 (75.5%) children ≤ 5 years of age, 100.0% of them had received the Bacillus Calmette–Guérin (BCG) vaccine. Among 27 children ≥ 2 months, 16 (59.2%) had received at least one dose of *S. pneumoniae* vaccine and 22 (81.5%) had received at least one dose *H. influenzae* vaccine.

Imaging was available for 171/257 (66.5%) patients (CT scan: 150/171, 87.7%; MRI (magnetic resonance imaging): 8/171, 4.7%; CT and MRI: 13/171, 7.6%). Results were abnormal in 120/171 (70.2%) and were generally non-specific, among which 40/120 (33.3%) were oedema, 23/120 (19.2%) were focal brain lesions (brain abscess, space occupying, and/or ring-enhancing lesions), and 17/120 (14.2%) hydrocephalus.

### Infectious aetiologies

#### Confirmed-infectious aetiologies

**Figure 1:**
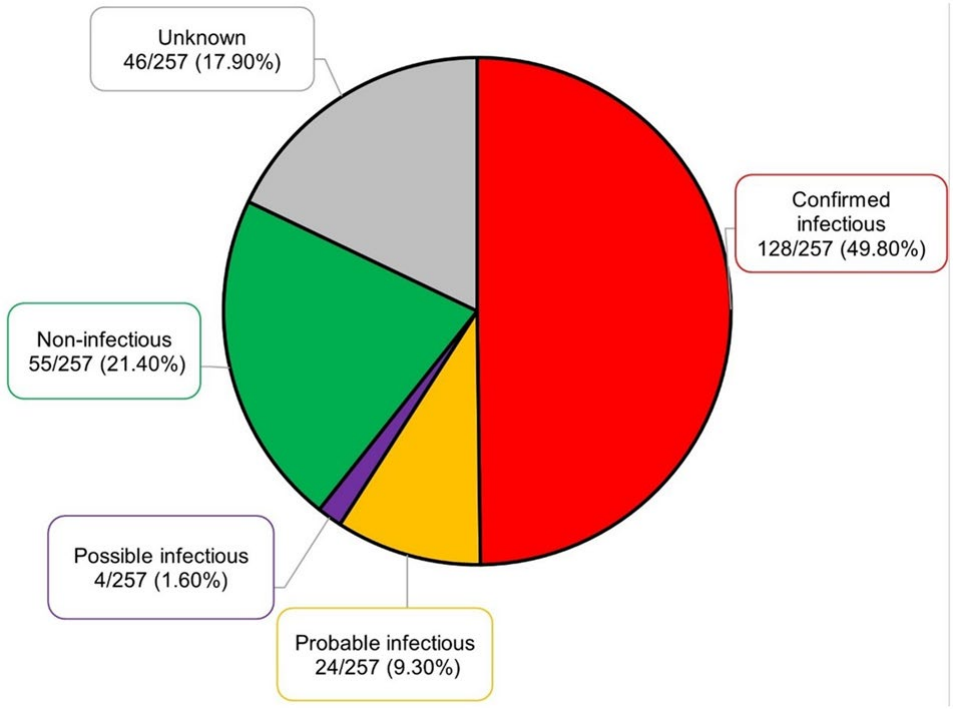
Distribution of patients with suspected central nervous system infection by confirmed, probable and possible infectious aetiology, non-infectious and unknown aetiology. Bolivia, 2017–2018.

The aetiological identification rate was 49.8% (128/257) (Fig. 1), more frequent in males (87/156, 55.8%) than females (41/101, 40.6%) (p = 0.017), but with no difference according to age-group (children 38/82, 46.4%; adults 90/175, 51.4%) or city (Santa Cruz 50/111, 45.0%; Cochabamba 78/146, 53.4%) (Table 2). Results of each diagnostic method are reported in the Supplementary Table S4.

**Table 2:** Demographics and HIV status of patients suspected of central nervous system infection by aetiologies. Bolivia, 2017–2018. Data are presented as number (%).

	Total	Male	Female		Children	Adults		HIV-positive	HIV-negative		SCZ	CBBA	
	n = 257	n = 156	n = 101		n = 82	n = 175		n = 52	n = 205		n = 111	n = 146	
	no. (%)	no. (%)	no. (%)	p-value	no. (%)	no. (%)	p-value	no. (%)	no. (%)	p-value	no. (%)	no. (%)	p-value
**CONFIRMED INFECTIOUS**	128 (49.8)	87 (55.8)	41 (40.6)	**0.017**	38 (46.4)	90 (51.4)	0.447	36 (69.2)	92 (44.9)	**0.002**	50 (45.0)	78 (53.4)	0.183
**Bacteria**	46 (17.9)	30 (19.2)	16 (15.8)	0.489	23 (28.0)	23 (13.1)	**0.004**	1 (1.9)	45 (22.0)	** < 0.001**	24 (21.6)	22 (15.2)	0.175
*Streptococcus pneumoniae*	15 (5.8)	10 (6.4)	5 (5.0)	0.626	7 (8.5)	8 (4.6)	0.206	0 (0.0)	15 (7.3)	**0.044**	7 (6.3)	8 (5.5)	0.779
*Neisseria meningitidis*	2 (0.8)	1 (0.6)	1 (1.0)	0.756	0 (0.0)	2 (1.1)	0.331	0 (0.0)	2 (1.0)	0.474	1 (0.9)	1 (0.7)	0.845
*Haemophilus influenzae* type b	1 (0.4)	1 (0.6)	0 (0.0)	0.42	1 (1.2)	0 (0.0)	0.143	0 (0.0)	1 (0.5)	0.614	1 (0.9)	0 (0.0)	0.25
*Listeria monocytogenes*	3 (1.2)	2 (1.3)	1 (1.0)	0.831	0 (0.0)	3 (1.7)	0.233	1 (1.9)	2 (1.0)	0.569	1 (0.9)	2 (1.4)	0.729
*Treponema pallidum*	1 (0.4)	0 (0.0)	1 (1.0)	0.213	1 (1.2)	0 (0.0)	0.143	0 (0.0)	1 (0.5)	0.614	1 (0.9)	0 (0.0)	0.25
Other bacteria^a^	24 (9.3)	16 (10.3)	8 (7.9)	0.529	14 (17.1)	10 (5.7)	**0.003**	0 (0.0)	24 (11.7)	**0.009**	13 (11.7)	11 (7.6)	0.254
***Mycobacteria***	33 (12.9)	23 (14.7)	10 (9.9)	0.257	4 (4.9)	29 (16.6)	**0.012**	8 (15.4)	25 (12.1)	0.539	8 (7.2)	25 (17.1)	**0.019**
*Mycobacterium tuberculosis*	29 (11.3)	20 (12.8)	9 (8.9)	0.333	3 (3.7)	26 (14.9)	**0.008**	8 (15.4)	21 (10.2)	0.295	7 (6.3)	22 (15.1)	**0.028**
*Mycobacterium* spp.	4 (1.6)	3 (1.9)	1 (1.0)	0.555	1 (1.2)	3 (1.7)	0.765	0 (0.0)	4 (1.9)	0.31	1 (0.9)	3 (2.0)	0.459
**Viruses**	14 (5.4)	9 (5.8)	5 (5.0)	0.777	7 (8.6)	7 (4.0)	0.135	1 (1.9)	13 (6.3)	0.209	9 (8.1)	5 (3.4)	0.101
Rabies virus	6 (2.3)	4 (2.6)	2 (2.0)	0.762	4 (4.9)	2 (1.1)	0.064	0 (0.0)	6 (2.9)	0.212	5 (4.5)	1 (0.7)	**0.044**
Varicella-zoster virus	6 (2.3)	4 (2.6)	2 (2.0)	0.762	3 (3.7)	3 (1.7)	0.335	1 (1.9)	5 (2.4)	0.826	3 (2.7)	3 (2.0)	0.733
Herpes simplex 1	1 (0.4)	1 (0.6)	0 (0.0)	0.42	0 (0.0)	1 (0.6)	0.492	0 (0.0)	1 (0.5)	0.614	1 (0.9)	0 (0.0)	0.25
Herpes simplex 2	1 (0.4)	0 (0.0)	1 (1.0)	0.213	0 (0.0)	1 (0.6)	0.492	0 (0.0)	1 (0.5)	0.614	0 (0.0)	1 (0.7)	0.382
**Fungi**	16 (6.2)	12 (7.7)	4 (4.0)	0.226	3 (3.7)	13 (7.4)	0.244	12 (23.1)	4 (2.0)	** < 0.001**	5 (4.5)	11 (7.5)	0.319
*Cryptococcus neoformans*	10 (3.9)	8 (5.1)	2 (2.0)	0.202	0 (0.0)	10 (5.7)	**0.027**	7 (13.5)	3 (1.5)	** < 0.001**	5 (4.5)	5 (3.4)	0.657
*Cryptococcus* spp.	6 (2.3)	4 (2.6)	2 (2.0)	0.762	3 (3.7)	3 (1.7)	0.335	5 (9.6)	1 (0.5)	** < 0.001**	0 (0.0)	6 (4.1)	**0.031**
**Parasites**	9 (3.5)	7 (4.5)	2 (2.0)	0.285	0 (0.0)	9 (5.1)	**0.037**	9 (17.3)	0 (0.0)	** < 0.001**	2 (1.8)	7 (4.7)	0.131
*Toxoplasma gondii*	5 (1.9)	4 (2.6)	1 (1.0)	0.372	0 (0.0)	5 (2.8)	0.122	5 (9.6)	0 (0.0)	** < 0.001**	2 (1.8)	3 (2.0)	0.884
*Trypanosoma cruzi*	3 (1.2)	2 (1.3)	1 (1.0)	0.831	0 (0.0)	3 (1.7)	0.233	3 (5.8)	0 (0.0)	** < 0.001**	0 (0.0)	3 (2.0)	0.129
*Taenia solium*	1 (0.4)	1 (0.6)	0 (0.0)	0.42	0 (0.0)	1 (0.6)	0.493	1 (1.9)	0 (0.0)	**0.047**	0 (0.0)	1 (0.7)	0.382
**Co-infections**	10 (3.9)	6 (3.8)	4 (4.0)	0.963	1 (1.2)	9 (5.1)	0.129	5 (9.6)	5 (2.5)	**0.017**	2 (1.8)	8 (5.5)	0.131
*S. pneumoniae* + Cytomegalovirus	1 (0.4)	1 (0.6)	0 (0.0)	0.42	1 (1.2)	0 (0.0)	0.143	0 (0.0)	1 (0.5)	0.614	1 (0.9)	0 (0.0)	0.25
*S. pneumoniae* + *Cryptococcus* spp.	1 (0.4)	1 (0.6)	0 (0.0)	0.42	0 (0.0)	1 (0.6)	0.493	0 (0.0)	1 (0.5)	0.614	0 (0.0)	1 (0.7)	0.382
*M. tuberculosis* + *C. neoformans*	1 (0.4)	1 (0.6)	0 (0.0)	0.42	0 (0.0)	1 (0.6)	0.493	1 (1.9)	0 (0.0)	**0.047**	0 (0.0)	1 (0.7)	0.382
*M. tuberculosis* + other bacteria^b^	4 (1.5)	2 (1.3)	2 (2.0)	0.659	0 (0.0)	4 (2.3)	0.168	1 (1.9)	3 (1.5)	0.811	0 (0.0)	4 (2.7)	0.078
*C. neoformans* + *Trypanosoma cruzi*	2 (0.8)	1 (0.6)	1 (1.0)	0.756	0 (0.0)	2 (1.1)	0.331	2 (3.9)	0 (0.0)	**0.005**	1 (0.9)	1 (0.7)	0.845
*T. gondii* + Epstein-Barr	1 (0.4)	0 (0.0)	1 (1.0)	0.213	0 (0.0)	1 (0.6)	0.493	1 (1.9)	0 (0.0)	**0.047**	0 (0.0)	1 (0.7)	0.382
**PROBABLE AND POSSIBLE INFECTIOUS**	28 (10.9)	14 (9.0)	14 (13.9)	0.219	6 (7.3)	22 (12.6)	0.208	10 (19.2)	18 (8.8)	**0.031**	10 (9.0)	18 (12.4)	0.397
**NON-INFECTIOUS**	55 (21.4)	28 (17.9)	27 (26.7)	0.093	21 (25.6)	34 (19.4)	0.26	0 (0.0)	55 (26.8)	** < 0.001**	24 (21.6)	31 (21.2)	0.94
Anti-NMDAR	3 (1.2)	1 (0.6)	2 (2.0)	0.329	2 (2.4)	1 (0.6)	0.194	0 (0.0)	3 (1.5)	0.38	2 (1.8)	1 (0.7)	0.409
**UNKNOWN**	46 (17.9)	27 (17.3)	19 (18.8)	0.759	17 (20.7)	29 (16.6)	0.417	6 (11.6)	40 (19.5)	0.18	27 (24.4)	19 (13.0)	**0.019**

^*a*^*K. pneumoniae* (n = 6)*, A. baumannii* (n = 4)*, P. aeruginosa* (n = 2)*, S. aureus* (n = 2), *Streptococcus* spp. (n = 2), *E. coli* (n = 2)*, S. pyogenes* (n = 1)*, E. faecium* (n = 1) *S. epidermidis* (n = 1)*, Achromobacter* spp. (n = 1), *S. maltophilia* (n = 1), *Staphylococcus* spp. (n = 1); ^b^*Acinetobacter* spp. (n = 1), *Burkholderia* spp. (n = 1)*, Pseudomona* spp. (n = 1), *S. epidermidis* (n = 1).

Abbreviations: SCZ, Santa Cruz; CBBA, Cochabamba; Anti-NMDAR, *N*-Methyl-D-aspartic acid receptor.

Significant values are in [bold]

#### Confirmed-infectious aetiologies according to HIV status

Among confirmed infectious aetiologies (n = 128), the most frequent pathogens were: (i) in HIV-positive patients, *Cryptococcus* spp. (12/36, 33.3%), *Mycobacterium tuberculosis* (8/36, 22.2%), *Toxoplasma gondii* (5/36, 13.9%), *Trypanosoma cruzi* (3/36, 8.3%), and co-infections (5/36, 13.9%; including *M. tuberculosis*, *Cryptococcus* spp. and *Trypanosoma cruzi*); (ii) in HIV-negative patients, *Mycobacterium tuberculosis* (21/92, 22.8%), *Streptococcus pneumoniae* (15/92, 16.3%), rabies (6/92, 6.5%), varicella-zoster (5/92, 5.4%), and co-infections (5/92, 5.4%; including mainly *M. tuberculosis*).

**Figure 2:**
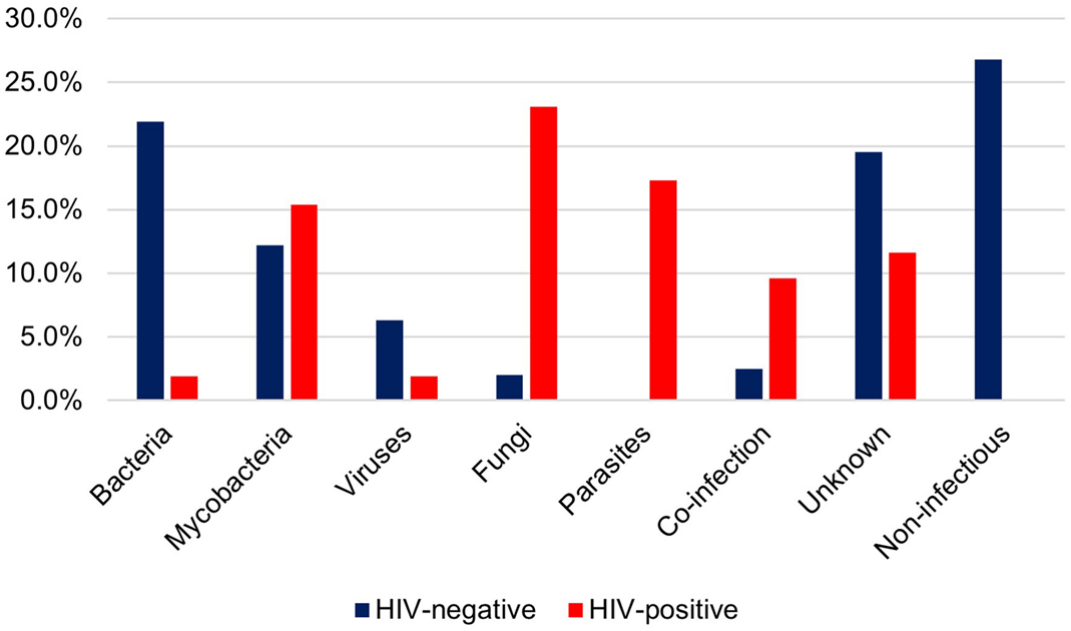
Aetiologies distribution of patients with suspected central nervous system infection by HIV status. Bolivia 2017–2018.

Bacterial infections were more frequent in HIV-negative (p < 0.001), and more fungal (p < 0.001), parasitic (p < 0.001), and co-infections (p = 0.017) in HIV-positive patients (Fig. 2).

#### Confirmed-infectious aetiologies according to age-group

Among confirmed single and co-infections, the most common pathogen was *Streptococcus pneumoniae* (8/38, 21.0%) in children, and *Mycobacterium tuberculosis* (31/90, 34.4%) in adults.

#### Confirmed-infectious aetiologies according to region

Mycobacterial infections were more frequently found in Cochabamba (25 of 33 cases), and more rabies cases (5 of 6 cases) in Santa Cruz.

#### Clinical features, blood, and CSF parameters in confirmed-infectious aetiologies

Fever (91.4%), headache (67.2%), neck stiffness (55.5%), and GCS < 12 (42.5%) were more frequently associated with confirmed-infectious than with unknown and non-infectious aetiologies. More HIV-negative than HIV-positive patients had fever (95.7% *vs* 80.5%) (p = 0.006). Clinical features were poorly pathogen specific (Supplementary Table S6), except for rabies (hydrophobia, aerophobia, photophobia). Among blood parameters (Supplementary Table S7), hyperglycaemia was associated with bacterial infections (p < 0.001).

Twenty-two (8.6%) confirmed aetiologies had normal CSF parameters (8/14, 57.1% in case of viral aetiology). Bacterial infections were associated with opalescent or turbid appearance with a higher proportion of neutrophils in the CSF (p < 0.001). Xanthochromia in CSF was frequently associated with mycobacterial infections (86.0%). In HIV-positive patients, *Cryptococcus* spp. and mycobacteria showed similar CSF findings (elevated white cell count (WBC) with lymphocytic pleocytosis, and low % CSF/serum glucose (median ~ 20)). However, normal proteins were predominantly suggestive of *Cryptococcus* spp. (p = 0.033). Similar CSF characteristics (clear, WBC < 100/mm^3^, proteins < 150 mg/dL, and normal glucose) were frequently found in both parasitic and viral infections, but the aetiological identification varied according to the HIV status (Supplementary Table S8, Supplementary Figure S1).

### Probable and possible infectious aetiologies

Twenty-four (9.3%) cases were classified as probable and 4 (1.6%) as possible aetiologies. In HIV-positive patients, *Toxoplasma gondii* (4/52, 7.7%), *Mycobacterium tuberculosis* (2/52, 3.8%), *Cryptococcus* spp. (1/52, 1.9%), HIV (1/52, 1.9%), JC virus (1/52, 1.9%) represented probable aetiologies, and influenza B (1/52, 1.9%) a possible aetiology. In HIV-negative patients, M*ycobacterium tuberculosis* (1/205, 0.5%), Zika virus (1/205, 0.5%), other bacteria (13/205, 6.3%) represented probable aetiologies, and influenza B (2/205, 1.0%) and cytomegalovirus (1/205, 0.5%) possible aetiologies.

Respiratory pathogens were identified in 34/200 (17.0%) nasopharyngeal swabs, more frequently in children (18/57, 31.6%) than adults (16/161, 9.9%) (p < 0.001), but not differently in those with or without an infectious aetiology (Supplementary Table S9).

### Non-infectious and unknown aetiologies.

Fifty-five (21.4%) patients had a final clinical diagnosis of non-infectious aetiology, and 46 (17.9%) remained without any final diagnosis. Three patients with antibodies against N-methyl-D-aspartate receptor (NMDAR) were classified as confirmed autoimmune encephalitis, all of them were under the age of 17. Vascular encephalopathy (11, 20.0%), and epilepsy (8, 14.5%) were the most common non-infectious aetiologies. In addition, one case of Guillain-Barré syndrome had serum IgM antibodies to Zika virus.

CSF parameters were normal in 41 of 51 (74.5%) non-infectious aetiologies and in 25 of 46 (54.3%) unknown aetiologies (Supplementary Figure S1). Among abnormal CSF in unknown aetiologies (21/46; 45.7%), 5/21 (23.8%) showed CSF characteristics similar to mycobacterial infections.

### Clinical syndromes

208/257 (80.9%) patients met the WHO criteria for CNS infection. Of all infectious confirmed cases, 16/128 (12.5%) did not fulfil these criteria (Supplementary Table S10), with 11 patients without fever (7 HIV-positive), and 5 who had only fever. Of note, 6 were HIV-positive patients with focal brain lesions. More HIV-positive than HIV-negative patients with a confirmed-infectious aetiology (25.0% *vs* 7.6%) did not fulfil the WHO criteria (p = 0.007). All parasitic infections presented with focal brain lesions.

### Treatment

Two hundred thirty-three (90.7%) patients received empirical treatment with third-generation cephalosporins during hospitalization. 48/257 (18.7%) patients received acyclovir, including the eight patients with herpes or varicella-zoster infection (7/8 were treated before lumbar puncture). 71/257 (27.6%) patients received tuberculosis management, including 31/33 (93.9%) of those with proven mycobacterial infection. In HIV-negative patients with both xanthochromia in CSF and a meningoencephalitis, 23/27 (85.0%) had a biological diagnosis of mycobacterial infection, and four remained without any aetiological diagnosis (may include undiagnosed mycobacterial infections). This specific association was therefore highly predictive of mycobacterial infection. However, the same association did not increase the predictability (71.0%) among HIV-positive patients. 44/257 (17.1%) patients received antifungal medication. Among patients with cryptococcal infection, 60.0% received treatment with fluconazole, and 40.0% with fluconazole combined with amphotericin B.

### Risk factors for severity

#### Mortality

The overall rate was 42.1% (94/223): 63 (28.2%) died during hospitalization, 24 (10.8%) in the first three months of follow-up, and 7 (3.1%) during the next three months. 34 (13.2%) patients were lost during follow-up and excluded from statistics. In multivariate analysis, death was associated with thrombocytopenia (Odds ratio (OR) 5.40, 95% CI 2.40–11.83) and hydrocephalus (OR 4.07, 95% CI 1.35–12.23) (Supplementary Table S11 and S12).

**Figure 3:**
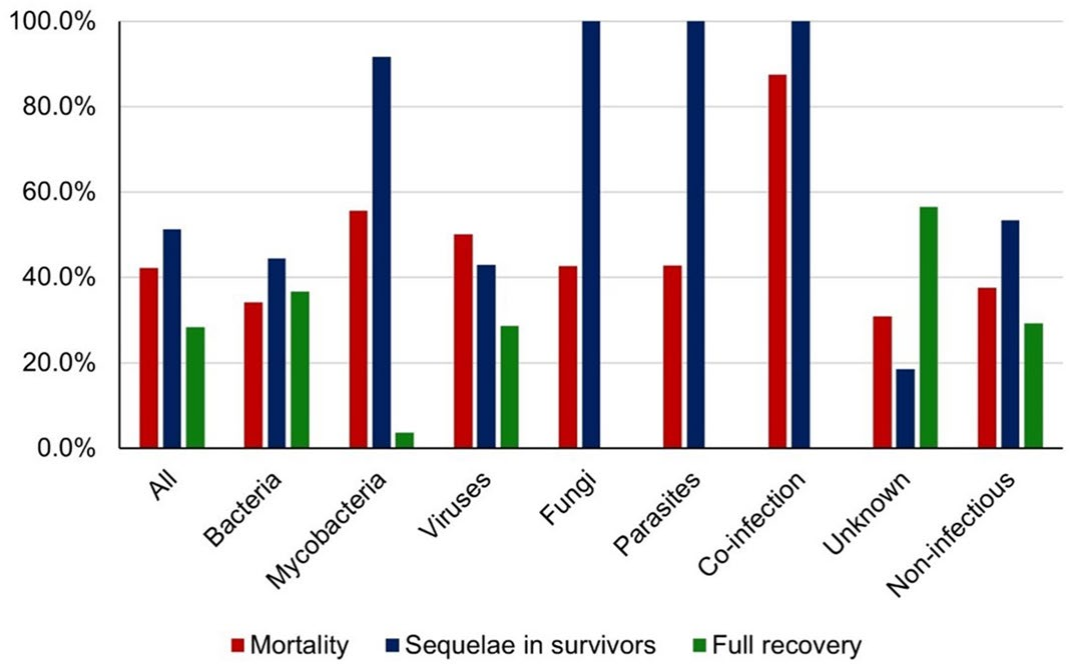
Clinical outcome of patients with suspected central nervous system infection by aetiology. Bolivia 2017–2018.

Mortality in patients with an infectious aetiology (52/110, 47.2%) was (non-significantly) higher than in those with a non-infectious (18/48, 37.5%) or an unknown aetiology (12/39, 30.8%). It was 54.2% (26/48) in HIV-positive and 38.9% (68/175) in HIV-negative patients. Mortality according to microorganism class was: co-infections 7/8, 87.5%; mycobacteria 15/27, 55.5%; viruses 7/14, 50.0% (6/7 due to rabies); fungi 6/13, 46.2%; parasites 3/7, 42.8%; bacteria 14/41, 34.1% (Fig. 3).

Mortality among patients receiving cryptococcal treatment was 25.0% in those treated by amphotericin B and fluconazole, and 87.5% in those who received fluconazole only (p = 0.040).

#### Sequelae in survivors

In those alive at the end of follow-up, neurological sequelae included coma, motor deficits (specifically in *M. tuberculosis*), cognitive impairment, hearing loss (specifically in *Cryptococcus* spp.), blindness, unresolved headache, secondary epilepsy, dysarthria, and amnesia.

The global sequelae rate was 51.2% (66/129). It was higher in HIV-positive (17/22, 77.3%) than HIV-negative patients (49/107, 45.8%) (p = 0.007). In multivariate analysis, sequelae were associated with HIV-positive patients (OR 4.0, 95% CI 1.3–11.7), and mycobacterial infection (OR 12.4, 95% CI 1.5–99.1) (Supplementary Table S13 and S14).

Long-term sequelae rates according to microorganism class were: fungal (7/7), parasitic (4/4), and co-infections (4/4), 100.0%; mycobacteria 11/12, 91.7%; bacteria 12/27, 44.4%; viruses 3/7, 42.9% (Fig. 3).

## Discussion

This study represents an attempt to investigate CNS infections in Cochabamba and Santa Cruz, Bolivia. We included inpatients when the physician in charge suspected a CNS infection and performed a lumbar puncture. In a context of limited laboratory capacity, the study allowed access to first-line diagnosis of frequent or treatable pathogens performed in the time of care, and to a second-line of complementary diagnostics. An infectious aetiology was confirmed for 49.8% (128/257) of patients, and an autoimmune encephalitis (anti-NMDA receptor encephalitis) in 1.2% (3/257) of patients. These numbers are in the proportion of those obtained in previous studies including all types of CNS infection syndromes: Netherlands, 25.0%^28^; Nepal, 38.0%^1^; Laos, 42.3%^3^; Switzerland, 42.7%^29^; Georgia, 51.0%^30^; Vietnam, 52.0%^31^; Uganda, 54.9%^32^; Singapore, 55.3%^33^, 58.0%^34^; suggesting that diagnostic tests were meaningfully adapted to the local situation.

Our study had several limitations due to imaging (most of the CT scans were performed without contrast, MRI were performed in a few patients); biological diagnostics (second-line diagnosis) was not performed in all patients, and the collection of convalescent serum specimen was not possible due to the difficulty of following up patients (e.g. transfer to another medical department, early discharge); some pathogens (*e.g. M. tuberculosis*, *T. gondii*, *T. solium*) may have been missed due to suboptimal methods; additional assays and deep sequencing may have improved aetiological resolution. In addition, we included patients from two departments of Bolivia, more adults than children, and more patients from tertiary-level public hospitals, than private or primary and secondary-level healthcare centers. This may have resulted in recruitment biases and combined with the specific Bolivian care pathway, suggests a probable selection of severe and late cases in a disadvantaged fraction of the population. To our best knowledge, this study is the first prospective investigation of CNS infections in Bolivia and represents a valuable contribution concerning Bolivian health policy. However, further studies across the country are needed to have a better understanding of each department situation.

Some of our observations strengthen previous observations made in different settings: (i) According to patients' clinical presentation, 12.5% of cases with a confirmed-infectious aetiology did not fulfil the WHO criteria for CNS infection (reaching 25.0% in HIV-positive patients) mainly due to the absence of fever. Coming after other similar reports^3^, this pleads for an improved standardized clinical case definition of CNS infections. (ii) As previously observed hyperglycaemia at admission was associated with bacterial infections^3,35^, of which 25.0% were detected in known diabetic patients; in other cases, we could not distinguish between pre-existing unrecognized diabetes and stress reaction leading to disturbed glycaemia regulation^35^. CSF findings cannot discriminate between aetiologies, but some predictors may help differentiate between groups (e.g. neutrophils in bacteria, xanthochromia in mycobacteria in HIV-negative patients). 54.3% of patients with unknown aetiologies had normal CSF findings; according to our data (57.1% in viral infections), some unidentified cases could then have been viral infections^33^. Deep investigations are required to identify the aetiology. (iii) The etiological pattern differed according to HIV status, including more bacterial infections in HIV-negative patients and more fungal, parasitic, and co-infections in HIV-positive patients^36^. Of note, enteroviruses, which are a common cause of CNS infections in high-income settings, were not detected. The absence of this pathogen in our study population could be explained by the fact that enteroviral meningitis is usually less severe, and the clinical manifestations in children are usually nonspecific^33,37–39^, therefore, patients might not have reached tertiary hospitals or have been misdiagnosed.

The overall case fatality rate in our series was high (42.1%). It was 28.2% during hospitalization, but one-third of deceased patients died within six months after discharge, indicating that a correct assessment of mortality requires a long follow-up and that circumstances of death in discharged patients deserve more attention in future studies. Neither the HIV status nor the diagnosis of tuberculosis were statistical independent drivers of mortality, but the co-infection by *M. tuberculosis* and HIV had a terrible mortality (83.3%). In multivariate analysis, hydrocephalus was a predictor of death (58.3% were observed in patients with tuberculosis among whom it represents a severity marker)^40,41^. It was also noted that thrombocytopenia, a biomarker of sepsis severity^42^, was significantly associated with mortality in multivariate analysis^43^.

The global analysis of patients' management revealed both strengths and weaknesses. Access to neuroimaging was suboptimal and a better access in the Bolivian health insurance would represent a sound step forward. Children immunization cards suggests that some vaccine-preventable bacterial infections (*S. pneumoniae* and *H. influenzae*) could have been avoided. By contrast, despite of the late arrival of many patients, ceftriaxone was given systematically and pre-emptively at inclusion, which conforms to international recommendations, and probably allowed saving lives. However, more efforts are needed to improve children vaccine coverage, and as recommended by the CDC, pneumococcal polysaccharide vaccine (PPSV23) should also be recommended for adults older than 65 years.

To conclude, three items in our study require specific attention in a public health perspective. First, one disquieting finding is the number of HIV-positive patients (20.2%) and the proportion of those in the last-stages of infection (86.7% AIDS), especially in Cochabamba –in a country where the prevalence of infection is low (0.3%). *Cryptococcus* spp. infection was the most frequent pathogen (41.7%). Patients may benefit from a more cost-effective and sensitive cryptococcal diagnostic test using the IMMY CrAg Lateral Flow Assay^44,45^, and improved CNS fungal clearance and a better therapeutic outcome by using a fluconazole/amphotericin B combination rather than fluconazole only^46,47^. In Bolivia, amphotericin B is not covered by the medical insurance and facilitating access to bi-therapy would represent an important issue. Finally, HIV-patients in our series represented probably patients with no access to antiviral therapy or who stopped it in a context of social stigmatisation and insufficient support. Obviously, the most effective strategy for preventing opportunistic infections in HIV-patients relies in the upstream improvement of the detection, management, and follow-up of HIV-infected patients. Second, *M. tuberculosis* represents a major public health problem in Bolivia^12^. Among confirmed single and co-infections, neurotuberculosis represented a prominent pathogen in both HIV-negative (26.1%) and –positive (27.8%) patients, and a significant difference was observed between children (7.9%) and adults (34.4%) (p = 0.002) (8.8% and 37.5%, respectively in HIV-negative patients), which may be attributed to BCG immunization coverage (100.0%), that could have limited the number of neurotuberculosis in children. We underline two challenges: diagnosis of tuberculosis deserves improvement by using the Xpert MTB/RIF Ultra assay, which showed higher sensitivity, as previously reported^48,49^; 72.4% of the total neurotuberculosis patients had preexisting known tuberculosis, chronic cough, or contact with family members with active pulmonary tuberculosis. An early treatment and follow-up until completion of treatment for non-neurological tuberculosis may limit the number of cases. Of note the association of xanthochromia in the CSF and meningoencephalitis in HIV-negative was predictive of neurotuberculosis and should expedite implementation of treatment^50^. *Third*, rabies is actively circulating in dogs in Bolivia. The number of reported cases in humans is low (~ two cases per year between 2011–2016)^51^, but our results suggest that it is under-valued, probably because of limited access to diagnosis. Over a one-year period in two sites only, rabies was responsible for six fatal cases in our study, occurring mainly in Santa Cruz. All ante-mortem cases were identified by real-time PCR^52^. We conclude that improving the awareness of the population, obtaining the availability of vaccines, and post-exposure prophylaxis represents a major and urgent public health objective.

## Supplementary Information

Supplementary Information.

## Data Availability

The data that support the findings of the study are available within the article. Additional data is available upon request from the corresponding author.

## References

[1] Giri A (2013). Aetiologies of central nervous system infections in adults in Kathmandu, Nepal: a prospective hospital-based study. Sci. Rep..

[2] Lancaster E (2016). The Diagnosis and Treatment of Autoimmune Encephalitis. J. Clin. Neurol..

[3] Dubot-Pérès A (2019). Management of Central Nervous System Infections, Vientiane, Laos, 2003–2011. Emerg. Infect. Dis..

[4] Pobreza y Desigualdad. *INE* https://www.ine.gob.bo/index.php/estadisticas-economicas/encuestas-de-hogares/.

[5] Perfil de los Sistemas de Salud - Bolivia. 58.

[6] Vigilato MAN, Cosivi O, Knöbl T, Clavijo A, Silva HMT (2013). Rabies Update for Latin America and the Caribbean. Emerg Infect Dis.

[7] endemicity_dog_mediated_rabies_map_2016.jpg (1187×833). https://www.who.int/rabies/endemicity_dog_mediated_rabies_map_2016.jpg?ua=1.

[8] snis.minsalud.gob.bo - Inicio. https://snis.minsalud.gob.bo/.

[9] Guarachi Catari, B. Características de la neuroinfección en niños de 0 a 14 años de edad en el Hospital del Niño "Dr. Ovidio Aliaga Uría. *Revista de la Sociedad Boliviana de Pediatría* **50**, 70–74 (2011).

[10] Taboada Villca, J. P., Pedrozo Gómez, S. R. & Castro Soto, M. del R. MENINGOENCEFALITIS TUBERCULOSA EN EL HOSPITAL CLÍNICO FRANCISCO VIEDMA. 1 ° ENERO-2000 AL 31 DICIEMBRE - 2003 COCHABAMBA-BOLIVIA. *Revista Ciencia y Medicina* **6**, 67 (2005).

[11] Nicoletti A (2005). Epilepsy and neurocysticercosis in rural Bolivia: a population-based survey. Epilepsia.

[12] WHO | Global tuberculosis report 2019. https://www.who.int/tb/publications/global_report/en/.

[13] PAHO/WHO - SIREVA II. https://www.paho.org/hq/index.php?option=com_docman&view=list&slug=sireva-ii-8059&Itemid=270&lang=en.

[14] Pérez Miranda F (2010). Estudio en el grupo de meningitis bacteriana, influencia de la malnutrición en el curso de niños con meningitis bacteriana. Revista de la Sociedad Boliviana de Pediatría.

[15] Gabastou, J.-M. *et al.* Caracterización de aislamientos invasivos de S. pneumoniae, H. influenzae y N. meningitidis en América Latina y el Caribe: SIREVA II, 2000–2005. *Rev Panam Salud Publica* **24**, 1–15 (2008). 10.1590/s1020-49892008000700001 18764989

[16] Organization, W. H. Progress introducing Haemophilus influenzae type b vaccine in low-income countries, 2004–2008 = Progrès de l’introduction du vaccin anti-Haemophilus influenzae type b dans les pays à faible revenu, 2004–2008. *Weekly Epidemiological Record = Relevé épidémiologique hebdomadaire* **83**, 62–67 (2008). 18283719

[17] Ministerio de Salud y Deportes de Bolivia - 150000 Niños Recibieron la Vacuna Contra el Neumococo. https://www.minsalud.gob.bo/91-150-000-ninos-recibieron-la-vacuna-contra-el-neumococo.

[18] Number of people living with HIV. *Our World in Data* https://ourworldindata.org/grapher/number-of-people-living-with-hiv.

[19] Death rate from HIV/AIDS. *Our World in Data* https://ourworldindata.org/grapher/hiv-death-rates.

[20] Castro Soto, M. del R. & Córdova Arancibia, H. Características clínicas y laboratoriales de la coinfeccion VIH-SIDA y criptococosis meningea en el Hospital Clínico Viedma de Cochabamba, Bolivia. *Gaceta Médica Boliviana* **37**, 64–67 (2014).

[21] Torrico, F. & Castro Soto, M. del R. Co-infección por Trypanosoma Cruzi y VIH: reporte de un caso de meningoencefalitis chagásica en Cochabamba, Bolivia. *Gaceta Médica Boliviana* **36**, 96–99 (2013).

[22] Steinberg, H. E. *et al.* Detection of toxoplasmic encephalitis in HIV positive patients in urine with hydrogel nanoparticles. *PLoS Negl. Trop. Dis.* **15**, (2021). 10.1371/journal.pntd.0009199 PMC7954332 33651824

[23] Bolivia cuenta con más de 11 millones de habitantes a 2018. *INE* https://www.ine.gob.bo/index.php/bolivia-cuenta-con-mas-de-11-millones-de-habitantes-a-2018/ (2018).

[24] Brain Infections Group - Institute of Infection and Global Health - University of Liverpool. https://www.liverpool.ac.uk/infection-and-global-health/research/brain-infections-group/.

[25] Venkatesan A (2013). Case Definitions, Diagnostic Algorithms, and Priorities in Encephalitis: Consensus Statement of the International Encephalitis Consortium. Clin. Infect. Dis..

[26] Ninove, L. *et al.* RNA and DNA Bacteriophages as Molecular Diagnosis Controls in Clinical Virology: A Comprehensive Study of More than 45,000 Routine PCR Tests. *PLOS ONE* **6**, e16142 (2011). 10.1371/journal.pone.0016142 PMC3036576 21347398

[27] Thirion L (2020). Lyophilized Matrix Containing Ready-to-Use Primers and Probe Solution for Standardization of Real-Time PCR and RT-qPCR Diagnostics in Virology. Viruses.

[28] Khatib U, van de Beek D, Lees JA, Brouwer MC (2017). Adults with suspected central nervous system infection: A prospective study of diagnostic accuracy. J. Infect..

[29] Dittrich T (2020). Predictors of infectious meningitis or encephalitis: the yield of cerebrospinal fluid in a cross-sectional study. BMC Infect. Dis..

[30] Akhvlediani, T. *et al.* Etiologic Agents of Central Nervous System Infections among Febrile Hospitalized Patients in the Country of Georgia. *PLOS One* **9**, e111393 (2014). 10.1371/journal.pone.0111393 PMC4219716 25369023

[31] Trung, N. H. D. *et al.* Aetiologies of Central Nervous System Infection in Viet Nam: A Prospective Provincial Hospital-Based Descriptive Surveillance Study. *PLOS One* **7**, e37825 (2012). 10.1371/journal.pone.0037825 PMC3360608 22662232

[32] Page A-L (2017). Aetiology and Outcomes of Suspected Infections of the Central Nervous System in Children in Mbarara, Uganda.. Sci. Rep..

[33] Zellweger RM (2020). Disentangling etiologies of CNS infections in Singapore using multiple correspondence analysis and random forest. Sci. Rep..

[34] Wong SKS (2016). Epidemiology and etiology of suspected central nervous system infections in Singapore. Int. J. Infect. Dis..

[35] Schut ES (2009). Hyperglycemia in bacterial meningitis: a prospective cohort study. BMC Infect Dis.

[36] Archibald, L. K. & Quisling, R. G. Central Nervous System Infections. *Textbook of Neurointensive Care* 427–517 (2013) doi:10.1007/978-1-4471-5226-2_22.

[37] Mount HR, Boyle SD (2017). Aseptic and Bacterial Meningitis: Evaluation, Treatment, and Prevention. AFP.

[38] Kohil, A., Jemmieh, S., Smatti, M. K. & Yassine, H. M. Viral meningitis: an overview. *Arch. Virol.* 1–11 (2021) doi:10.1007/s00705-020-04891-1. 10.1007/s00705-020-04891-1 PMC7779091 33392820

[39] Jain S, Patel B, Bhatt GC (2014). Enteroviral encephalitis in children: clinical features, pathophysiology, and treatment advances. Pathog Glob Health.

[40] Raut T (2013). Hydrocephalus in tuberculous meningitis: Incidence, its predictive factors and impact on the prognosis. J. Infect..

[41] Chan KH (2003). Clinical relevance of hydrocephalus as a presenting feature of tuberculous meningitis. QJM.

[42] Greco E, Lupia E, Bosco O, Vizio B, Montrucchio G (2017). Platelets and Multi-Organ Failure in Sepsis. Int. J. Mol. Sci..

[43] Predictors of outcome in acute encephalitis - PubMed. https://pubmed.ncbi.nlm.nih.gov/23892708/. 10.1212/WNL.0b013e3182a2cc6d PMC3908458 23892708

[44] Cáceres, D. H. *et al.* Evaluation of a Cryptococcal antigen Lateral Flow Assay in serum and cerebrospinal fluid for rapid diagnosis of cryptococcosis in Colombia. *Rev. Inst. Med. Trop. Sao Paulo* **59**, e76 (2017). 10.1590/S1678-9946201759076 PMC5738761 29267584

[45] Rajasingham R (2017). Global burden of disease of HIV-associated cryptococcal meningitis: an updated analysis. Lancet Infect Dis.

[46] Santos JRA (2017). High-dose fluconazole in combination with amphotericin B is more efficient than monotherapy in murine model of cryptococcosis. Sci Rep.

[47] Larsen RA, Bauer M, Thomas AM, Graybill JR (2004). Amphotericin B and Fluconazole, a Potent Combination Therapy for Cryptococcal Meningitis. Antimicrob. Agents Chemother..

[48] Opota O, Mazza-Stalder J, Greub G, Jaton K (2019). The rapid molecular test Xpert MTB/RIF ultra: towards improved tuberculosis diagnosis and rifampicin resistance detection. Clin. Microbiol. Infect..

[49] Sekyere JO, Maphalala N, Malinga LA, Mbelle NM, Maningi NE (2019). A Comparative Evaluation of the New Genexpert MTB/RIF Ultra and other Rapid Diagnostic Assays for Detecting Tuberculosis in Pulmonary and Extra Pulmonary Specimens. Sci. Rep..

[50] Marcos, V. M. & Etessam, J. P. *Meningitis, encefalitis y otras infecciones del SNC*. (Elsevier Health Sciences Spain, 2014).

[51] Revista Epidemiologica. https://www.minsalud.gob.bo/boletin-informativo/1522-revista-epidemiologica.

[52] Wakeley PR (2005). Development of a Real-Time, TaqMan Reverse Transcription-PCR Assay for Detection and Differentiation of Lyssavirus Genotypes 1, 5, and 6. J Clin Microbiol.

