# Supplementary material for: A clinical, aetiological, and public health perspective on central nervous system infections in Bolivia, 2017–2018
Source: Sci Rep. 2021 Dec 1;11:23235. doi: 10.1038/s41598-021-02592-6 (PMC8636643; doi:10.1038/s41598-021-02592-6)
Supplement: Supplementary file 1 — Supplementary Information. [file 41598_2021_2592_MOESM1_ESM.pdf]

SUPPLEMENTARY DATA

A clinical, aetiological, and public health perspective on central nervous system infections in Bolivia, 2017-2018

Authors: Paola Mariela Saba Villarroel, María del Rosario Castro Soto, Oriana Melendres Flores, Alejandro Peralta Landívar, María E. Calderón, Roxana Loayza, José Boucraut, Laurence Thirion, Audrey Dubot-Pérès, Laetitia Ninove, Xavier de Lamballerie

Health care center	Level of care	Status	Recruitment	Total inclusions n=257 no. (%)
**COCHABAMBA**				
Viedma Hosp.	Tertiary	Public	Adults	105 (40.8)
M. Ascencio Villarroel Hospital	Tertiary	Public	Children	20 (7.8)
Los Olivos Clinic	Tertiary	Private	Adults	7 (2.7)
Univalle Hospital	Tertiary	Private	Adults	6 (2.3)
Copacabana Clinic	Tertiary	Private	Adults	2 (0.8)
Caja Petrolera de Salud Hospital	Tertiary	Social security	Adults	2 (0.8)
San Vicente Hospital	Tertiary	Private	Children	1 (0.4)
San Pedro Clinic	Tertiary	Private	Adults	1 (0.4)
María de los Ángeles Clinic	Tertiary	Private	Adults	1 (0.4)
Los Ángeles Clinic	Tertiary	Private	Adults	1 (0.4)
**SANTA CRUZ**				
Mario Ortíz Suárez Hospital	Tertiary	Public	Children	51 (19.8)
Japonés Hospital	Tertiary	Public	Children and adults	42 (16.3)
San Juan de Dios Hospital	Tertiary	Public	Adults	11 (4.3)
Francés Hospital	Secondary	Public	Children and adults	3 (1.2)
Bajío del Oriente Hospital	Secondary	Public	Adult	1 (0.4)
Foianini Clinic	Tertiary	Private	Adult	1 (0.4)
Nuclear Clinic	Tertiary services	Private	Children	1 (0.4)
Niño Jesús Clinic	Tertiary	Private	Children	1 (0.4)

Supplementary Table S1. Recruited patients with suspected central nervous system infections by healthcare center. Bolivia, 2017-2018. All tertiary-level public healthcare centers from Cochabamba and Santa Cruz were included, except for the Maternity, Institute of Gastroenterology, and Psychiatric hospital in Cochabamba, and the Maternity and Oncology hospital in Santa Cruz.

First prospective line

HIV-positive & HIV-negative patients		
**Pathogen**	**Sample**	**Test**
Cryptococcus spp**.**	CSF	Latex
N. meningitidis	CSF	qPCR
S. pneumoniae	CSF	qPCR
H. influenzae	CSF	qPCR
L. monocytogenes	CSF	qPCR
M. tuberculosis	CSF	qPCR
Leptospira spp.	CSF	qPCR
Herpes simplex	CSF	qPCR
Varicella-zoster	CSF	qPCR
Cytomegalovirus	CSF	qPCR
Enterovirus	CSF	qRT-PCR
Zika virus	CSF	qRT-PCR
Dengue virus	CSF	qRT-PCR
Mumps	CSF	qRT-PCR
HIV-positive patients		
Epstein-Barr	CSF	qPCR
JC virus	CSF	qPCR
Toxoplasma gondii	CSF	qPCR
Trypanosoma cruzi	CSF	qPCR
Taenia solium	CSF	qPCR

Second retrospective line

HIV-positive & HIV-negative patients		
**Pathogen**	**Sample**	**Test**
16S	CSF	PCR + seq
Mycobacterium spp.	CSF	qPCR
Rickettsia spp.	CSF	qPCR
Brucella spp.	CSF	qPCR
EEEV	CSF	qRT-PCR
SLEV	CSF	qRT-PCR
WEEV	CSF	qRT-PCR
VEEV	CSF	qRT-PCR
Measles	CSF	qRT-PCR
West Nile virus	CSF	qRT-PCR
Zika virus	Serum	qRT-PCR
Dengue virus	Serum	qRT-PCR
Chikungunya virus	Serum	qRT-PCR
Yellow fever virus	Serum	qRT-PCR
Bartonella spp.	Blood	qPCR
M. tuberculosis	CSF	Xpert MTB/RIF
M. tuberculosis	CSF	Xpert MTB/RIF Ultra
HIV-positive patients		
JC virus	Serum	qPCR
Epstein-Barr	Blood	qPCR
Toxoplasma gondii	Blood	qPCR

Second retrospective line

HIV-positive & HIV-negative patients		
**Pathogen/Antibody**	**Sample**	**Test**
Zika virus	Serum	ELISA IgM, AM
Dengue virus	Serum	ELISA IgM
Chikungunya virus	Serum	ELISA IgM
Toxoplasma gondii	Serum	ELISA IgA, M, G
Cytomegalovirus	Serum	ELISA IgM
Taenia solium	CSF & serum	ELISA IgG & IB
Treponema pallidum	CSF	Immunoblot
Autoimmune	CSF & serum	IHC
Glutamate receptors	CSF	IIFT
Cryptococcus spp.	Serum	Latex
Varicella-zoster	Serum	ELISA IgM

First prospective line in case of suspicion or retrospective second line

HIV-positive & HIV-negative patients		
Rabies	CSF & saliva	qRT-PCR
S. agalactiae	CSF	qPCR
Treponema pallidum	CSF	qPCR
Cytomegalovirus	Blood	qPCR
HHV-6	Blood	qPCR
Respiratory pathogens FTD 21	Nasopharyngeal swab	qRT-PCR

Abbreviations: CSF, cerebrospinal fluid; qPCR, real-time polymerase chain reaction; PCR, conventional polymerase chain reaction; qRT-PCR, real-time reverse transcription; Seq, sequencing; EEEV, Eastern equine encephalitis Virus; SLEV, St. Louis encephalitis virus; WEEV, Western equine encephalitis virus; VEEV, Venezuelan equine encephalitis virus; HHV-6; Human herpesvirus 6; ELISA, Enzyme-linked immunosorbent assay; IHC, immunohistochemistry; IIFT, Indirect immunofluorescence test; IB, immunoblot; glutamate receptors: *N*-methyl-D-aspartate (NMDA), α-amino-3-hydroxy-5-methyl-4-isoxazolepropionic acid (AMPA), leucine-rich glioma inactivated 1 (LGI1), contactin associated protein 2 (CASPR2), gamma-aminobutyric-acid B (GABAB) receptors; FTD 21, Fast-Track respiratory 21 pathogens: Influenza A, influenza B, influenza AH1N1, rhinovirus, coronavirus 229E, OC43, HKU1, and NL63, parainfluenza 1, 2, 3 and 4, bocavirus, *M. pneumoniae*, human respiratory syncytial virus A/B, enterovirus, parechovirus, adenovirus, human metapneumovirus A/B.

Supplementary Table S2. First- and second-line diagnostic panel tested in patients with suspected central nervous system infections. Bolivia, 2017-2018

Test	Oligo	5'-3' sequence	Reference
S. pneumoniae qPCR	PF<br>PR<br>Probe	GCGATAGCTTTCTCCAAGTGG<br>TTAGCCAACAAATCGTTTACCG<br>FAM-CCCAGCAATTCAAGTGTTCGCCGA-BHQ	Mediannikov O. *et al.*, 2014[1]
N. meningitidis qPCR	PF<br>PR<br>Probe	GTTCAAGTGGTGGAAAGCGG<br>TTTTCCTCCAGCCGTTTGAC<br>FAM-CAGTTGGCGATGGCA-BHQ	Morel A-S. *et al.*, 2015[2]
Varicella-zoster virus qPCR	PF<br>PR<br>Probe	GGTTAAACGTTTGAATCCATCC<br>CAGCAGACTTTCTCGAACGT<br>FAM-ATGCCACCTTTACAGTTGGAGGAA-TAMRA	Bousbia S. *et al.*, 2012[3]
Herpes simplex 1/2 qPCR	PF<br>PR<br>Probe	CATCACCGACCCGGAGAGGGAC<br>GGGCCAGGCGCTTGTTGGTGTA<br>FAM-CCGCCGAACTGAGCAGACACCCGCGC-TAMRA	Bousbia S. *et al.*, 2012[3]
Cytomegalovirus qPCR	PF<br>PR<br>Probe	GCAGCCACGGGATCGTACT<br>GGCTTTTACCTCACACGAGCATT<br>6FAM-CGCGAGACCGTGGAACTGCG-TAMRA	Bousbia S. *et al.*, 2012[3]
H. influenzae[a] qPCR	PF<br>PR<br>Probe	ATGGCGGGAACATCAATGA<br>ACGCATAGGAGGGAAATGGTT<br>6FAM-CGGTAATTGGGATCCAT-MGB-NFQ	Meyler K. L. *et al.*, 2012[4]
L. monocytogenes qPCR	PF<br>PR<br>Probe	TTTCATCCATGGCACCACC<br>ATCCGCGTGTTTCTTTTCGA<br>FAM-CGCCTGCAAGTCCTAAGACGCCA-TAMRA	Le Monnier *et al.*, 2011[5]
Pan-dengue qPCR	PF<br>PR<br>Probe	AGGACYAGAGGTTAGAGGAGA<br>CGYTCTGTGCCTGGAWTGAT<br>FAM-ACAGCATATTGACGCTGGGARAGACC -TAMRA	Leparc-Goffart I. *et al.*, 2009[6]
Pan-enterovirus qPCR	PF<br>PR<br>Probe	GCTGCGYTGGCGGCC<br>GAAACACGGACACCCAAAGTAGT<br>FAM-CTCCGGCCCCTGAATGYGGCTAA-TAMRA	Tapparel C. *et al.*, 2009[7]
Mumps qPCR	PF<br>PR<br>Probe	TCTCACCCATAGCAGGGAGTTATAT<br>GTTAGACTTCGACAGTTTGCAACAA<br>AGGCGATTTGTAGCACTGGATGGAACA	Uchida K. *et al.*, 2005[8]
Zika virus qPCR	PF<br>PR<br>Probe	CTTGGAGTGCTTGTGATT<br>CTCCTCCAGTGTTCATTT<br>FAM-AAGAAGAGAATGACCACAAAGATCATC -TAMRA	Atieh T. *et al.*, 2016[9]
Leptospira spp. qPCR	PF<br>PR<br>Probe	AAGCATTACCGCTTGTGGTG<br>GAACTCCCATTTCAGCGAT<br>FAM-AAAGCCAGGACAAGCGCCG-BHQ	Bourhy P. *et al.*, 2011[10]
Mycobacterium spp. qPCR	PF<br>PR<br>Probe	GGGTGGGGTGTGGTGTTTGA<br>CAAGGCATCCACCATGCGC<br>6FAM- TGGATAGTGGTTGCGAGCATC	Morel A-S. *et al.*, 2015[2]
M. tuberculosis qPCR	PF<br>PR<br>Probe	GGGTGGGGTGTGGTGTTTGA<br>CAAGGCATCCACCATGCGC<br>6FAM- GCTAGCCGGCAGCGTATCCAT	Morel A.-S. *et al.*, 2015[2]
Toxoplasma gondii qPCR	PF<br>PR<br>Probe	TTCTTCCCAGACGTGGATTTCC<br>GACAGACAGCGAACAGAACAGA<br>6FAM-TGGTTCCGCCTCCTTCGTCCGTCG-TAMRA	*Not published*
Epstein-Barr qPCR	PF<br>PR<br>Probe	GGAACCTGGTCATCCTTTGC<br>ACGTGCATGGACCGGTTAAT<br>VIC-CGCAGGCACTCGTACTGCTCGCT-TAMRA	Gunson R.N. et al., 2009[11]
Trypanosoma cruzi qPCR	PF<br>PR	ASTCGGCTGATCGTTTTCGA<br>AATTCCTCCAAGCAGCGGATA	Piron M. *et al.*, 2007[12]

	Probe	6FAM-CACACACTGGACACCAA-MGB	
Taenia solium qPCR	PF	CAGGGTGTGACGTCATGG	Yera H. *et al.*, 2011[13]
	PR	AGGAGGCCAGTTGCCTAGC	
	Probe	6FAM-AGGCTGTCCTTTGCCGT-BHQ	
JC virus qPCR	PF	ATACAGTGCTTTGCCTGAACC	Mengelle C. *et al.*, 2011[14]
	PR	CAACTGAGCAATAGCACTACC	
	Probe	6FAM-ACTGGAGCTCCGGGGGCTGTA-TAMRA	
Rabies qPCR	PF	ATGTAACACCYCTACAATG	Wakeley P.R. *et al.*, 2005[15]
	PR	GCAGGGTAYTTRTACTCATA	
	Probe	6FAM-ACAAGATTGTATTCAAAGTCAATAATCAG-TAMRA	
	Probe	AACARGGTTGTTTTYAAGGTCCATAA	
	Probe	6FAM-ACARAATTGTCTTCAARGTCCATAATCAG-TAMRA	
Treponema pallidum qPCR	PF	GTCGAGACTGAAAAGGAGTGCA	Leslie D.E *et al.*, 2007[16]
	PR	GTGAGCGTCTCATCATTCCAAAG	
	Probe	6FAM- TGCTGTGCAGGATCCGGCATATGTCC	
	PF	GCGGTTGCACAGTGGGAG	Salazar J.C *et al.*, 2007[17]
	PR	CAGCATGGGCGACAGGAT	
	Probe	6FAM- TTGTGCTGAATTCTTCCGCGCG-TAMRA	
S. agalactiae qPCR	PF	ATTGCGTGCCAACCCTGAG	Morel A-S *et al.*, 2015[2]
	PR	AAGGCTTCTACACGACTACCA	
	Probe	6FAM-CAGTTTATGATTTGAATTCT-MGB NFQ	
Eastern equine encephalitis qPCR	PF	TGTGCGTACCTCCTCATCGTT	Xiaoping Kang *et al.*, 2010[18]
	PR	GACTGGCGTGAATCTCTGCTT	
	Probe	VIC-AGCAGCCTACCTTTCCGACAATGGTTGTC-TAMRA	
St. Louis encephalitis qPCR	PF	CTGGCTGTCGGAGGGATTCT	Lanciotti *et al.*, 2001[19]
	PR	TAGGTCAATTGCACATCCCG	
	Probe	FAM-TCTGGCGACCAGCGTGCAAGCCG-TAMRA	
Venezuelan equine encephalitis virus	PF	TCCATGCTAATGCYAGAGCGTTTTCGCA	Vina-Rodriguez A., 2016[20]
	PR	TGGCGCACTTCCAATGTCHAGGAT	
	Probe	FAM-TGATCGARACGGAGGTRGAMCCATCC-TAMRA	
Western equine encephalitis qPCR	PF	AGGGATACCCCCGAAGGTT	Xiaoping Kang *et al.*, 2010[18]
	PR	GTGAATAGCACACGGGTGGTT	
	Probe	FAM-CTTTCGAATGTCACGTTCCCATGCG-TAMRA	
Rickettsia spp. qPCR	PF	GTGAATGAAAGATTACACTATTTAT	M. Varagnol *et al.*, 2009[21]
	PR	GTATCTTAGCAATCATTCTAATAGC	
	Probe	6FAM-CTATTATGCTTGCGGCTGTCGGTTC-TAMRA	
Measles qPCR	PF	TGGCATCTGAACTCGGTATCAC	Hummel K.B. *et al.*, 2006[22]
	PR	TGTCCTCAGTAGTATGCATTGCAA	
	Probe	6FAM-CCGAGGATGCAAGGCTTGTTTCAGA-TAMRA	
West Nile virus qPCR	PF	CCTGTGTGAGCTGACAAACTTAGT	Linke S *et al.*, 2007[23] Tang Y. *et al.*, 2006[24]
	PF	AAGTTGAGTAGACGGTGCTG	
	PR	GCGTTTTAGCATATTGACAGCC	
	PR	AGACGGTTCTGAGGGCTTAC	
	Probe	6FAM-CCTGGTTTCTTAGACATCGAGATCTTCGTGC-TAMRA	
	Probe	6FAM-CTCAACCCCAGGAGGACTGG-TAMRA	
Brucella spp. qPCR	PF	GCTCGGTTGCCAATATCAATGC	Probert W. *et al.*, 2004 [25]
	PR	GGGTAAAGCGTCGCCAGAAG	
	Probe	6FAM- AAATCTTCCACCTTGCCCTTGCCATCA	
Yellow fever qPCR	PF	AATCGAGTTGCTAGGCAATAAACAC	Drosten C. *et al.*, 2002[26]
	PR	TCCCTGAGCTTTACGACCAGA	
	Probe	6FAM-ATCGTTCGTTGAGCGATTAGCAG-TAMRA	
HHV6 qPCR	PF	CGCTAGGTTGAGRATGATCGA	Locatelli G. *et al.*, 2000[27]
	PR	CAAAGCCAAATTATCCAGAGCG	

	Probe	6FAM-CACCAGACGTCACACCCGAAGGAAT-TAMRA	
Bartonella spp. qPCR	PF	GATGCCGGGGAAGGTTTTC	Socolovschi C. *et al.*, 2012[28]
	PR	GCCTGGGAGGACTTGAACCT	
	Probe	6FAM-GCGCGCGCTTGATAAGCGTG-TAMRA	
Chikungunya qPCR	PF	AAGCTYCGCGTCCTTTACCAAG	Pastorino B. *et al.*, 2005[29]
	PR	CCAAATTGTCCYGGTCTTCCT	
	Probe	6FAM-CCAATGTCYTCMGCCTGGACACCTT-TAMRA	
T4 Phage qPCR	PF	CCATCCATAGAGAAAATATCAGAACGA	Ninove L. *et al.*, 2011[30]
	PR	CGCTGGGAAAAGAGGAATTATTTA	
	Probe	VIC-AACCAGTAATTTCATCTGCTTCTGATGTGAGGC-TAMRA	
MS2 phage qPCR	PF	CTCTGAGAGCGGCTCTATTGGT	Ninove L. *et al.*, 2011[30]
	PR	GTTCCCTACAACGAGCCTAAATTC	
	Probe	VIC-TCAGACACGCGGTCCGCTATAACGA-TAMRA	
Herpes simplex 1 qPCR	PF	CGGCCGTGTGACACTATCG	Weidmann M. *et al.*, 2003[31]
	PR	CTCGTAAAATGGCCCCTCC	
	Probe	CCATACCGACCACACCGACGAACC	
Herpes simplex 2 qPCR	PF	CGCTCTCGTAAATGCTTCCCT	Weidmann M. *et al.*, 2003[31]
	PR	TCTACCCACAACAGACCCACG	
	Probe	6FAM-CGCGGAGACATTCGAGTACCAGATCG-TAMRA	
Enterovirus EV71 qPCR	PF	GGAGAACACAARCARGAGAAAGA	Khanh TH., 2012[32]
	PR	ACTAAAGGGTACTTGGACTTVGA	
	Probe	6FAM-TGATGGGCACGTTCTCAGTGCG-BHQ1	
16S PCR	PF	CAGCAGCCGCGGTAATAC	Benslimani A *et al.*, 2005[33]
	PR	ACGGCTACCTTGTTACGACTT	
Abbreviations: PR, primer forward; PR, primer reverse. [a]H. influenzae type b tested using the kit Fast-Track diagnostics 33 pathogens (Fast-Track diagnostics, Luxemburg)			

Supplementary Table S3. List of primers and probes used to detect central nervous system pathogens by PCR. Bolivia, 2017-2018

References

1. Mediannikov, O. *et al.* Molecular identification of pathogenic bacteria in eschars from acute febrile patients, Senegal. *Am J Trop Med Hyg* **91**, 1015–1019 (2014).
2. Morel, A.-S. *et al.* Complementarity between targeted real-time specific PCR and conventional broad-range 16S rDNA PCR in the syndrome-driven diagnosis of infectious diseases. *Eur J Clin Microbiol Infect Dis* **34**, 561–570 (2015).
3. Bousbia, S. *et al.* Repertoire of Intensive Care Unit Pneumonia Microbiota. *PLOS ONE* **7**, e32486 (2012).
4. Meyler, K. L., Meehan, M., Bennett, D., Cunney, R. & Cafferkey, M. Development of a diagnostic real-time polymerase chain reaction assay for the detection of invasive Haemophilus influenzae in clinical samples. *Diagn Microbiol Infect Dis* **74**, 356–362 (2012).
5. Le Monnier, A., Abachin, E., Beretti, J.-L., Berche, P. & Kayal, S. Diagnosis of Listeria monocytogenes meningoencephalitis by real-time PCR for the hly gene. *J Clin Microbiol* **49**, 3917–3923 (2011).
6. Leparc-Goffart, I. *et al.* Development and validation of real-time one-step reverse transcription-PCR for the detection and typing of dengue viruses. *J Clin Virol* **45**, 61–66 (2009).
7. Tapparel, C. *et al.* New respiratory enterovirus and recombinant rhinoviruses among circulating picornaviruses. *Emerg Infect Dis* **15**, 719–726 (2009).
8. Uchida, K. *et al.* Rapid and sensitive detection of mumps virus RNA directly from clinical samples by real-time PCR. *J Med Virol* **75**, 470–474 (2005).
9. Atieh, T., Baronti, C., de Lamballerie, X. & Nougairède, A. Simple reverse genetics systems for Asian and African Zika viruses. *Scientific Reports* **6**, 39384 (2016).
10. Bourhy, P., Bremont, S., Zinini, F., Giry, C. & Picardeau, M. Comparison of real-time PCR assays for detection of pathogenic Leptospira spp. in blood and identification of variations in target sequences. *J Clin Microbiol* **49**, 2154–2160 (2011).

11. Gunson, R. N., Maclean, A. R., Shepherd, S. J. & Carman, W. F. Simultaneous detection and quantitation of cytomegalovirus, Epstein-Barr virus, and adenovirus by use of real-time PCR and pooled standards. *J Clin Microbiol* **47**, 765–770 (2009).
12. Piron, M. *et al.* Development of a real-time PCR assay for Trypanosoma cruzi detection in blood samples. *Acta Trop* **103**, 195–200 (2007).
13. Yera, H. *et al.* Confirmation and Follow-Up of Neurocysticercosis by Real-Time PCR in Cerebrospinal Fluid Samples of Patients Living in France. *J Clin Microbiol* **49**, 4338–4340 (2011).
14. Mengelle, C. *et al.* JC virus DNA in the peripheral blood of renal transplant patients: a 1-year prospective follow-up in France. *J Med Virol* **83**, 132–136 (2011).
15. Wakeley, P. R. *et al.* Development of a Real-Time, TaqMan Reverse Transcription-PCR Assay for Detection and Differentiation of Lyssavirus Genotypes 1, 5, and 6. *J Clin Microbiol* **43**, 2786–2792 (2005).
16. Leslie, D. E., Azzato, F., Karapanagiotidis, T., Leydon, J. & Fyfe, J. Development of a real-time PCR assay to detect Treponema pallidum in clinical specimens and assessment of the assay's performance by comparison with serological testing. *J Clin Microbiol* **45**, 93–96 (2007).
17. Salazar, J. C., Rathi, A., Michael, N. L., Radolf, J. D. & Jagodzinski, L. L. Assessment of the Kinetics of Treponema pallidum Dissemination into Blood and Tissues in Experimental Syphilis by Real-Time Quantitative PCR. *Infect Immun* **75**, 2954–2958 (2007).
18. Kang, X. *et al.* A duplex real-time reverse transcriptase polymerase chain reaction assay for detecting western equine and eastern equine encephalitis viruses. *Virol J* **7**, 284 (2010).
19. Lanciotti, R. S. & Kerst, A. J. Nucleic acid sequence-based amplification assays for rapid detection of West Nile and St. Louis encephalitis viruses. *J Clin Microbiol* **39**, 4506–4513 (2001).
20. Vina-Rodriguez, A., Eiden, M., Keller, M., Hinrichs, W. & Groschup, M. H. A Quantitative Real-Time RT-PCR Assay for the Detection of Venezuelan equine encephalitis virus Utilizing a Universal Alphavirus Control RNA. *Biomed Res Int* **2016**, 8543204 (2016).

21. Varagnol, M. *et al.* First detection of Rickettsia felis and Bartonella clarridgeiae in fleas from Laos. *Clin Microbiol Infect* **15 Suppl 2**, 334–335 (2009).
22. Hummel, K. B., Lowe, L., Bellini, W. J. & Rota, P. A. Development of quantitative gene-specific real-time RT-PCR assays for the detection of measles virus in clinical specimens. *J Virol Methods* **132**, 166–173 (2006).
23. Linke, S., Ellerbrok, H., Niedrig, M., Nitsche, A. & Pauli, G. Detection of West Nile virus lineages 1 and 2 by real-time PCR. *J Virol Methods* **146**, 355–358 (2007).
24. Tang, Y., Anne Hapip, C., Liu, B. & Fang, C. T. Highly sensitive TaqMan RT-PCR assay for detection and quantification of both lineages of West Nile virus RNA. *J Clin Virol* **36**, 177–182 (2006).
25. Probert, W. S., Schrader, K. N., Khuong, N. Y., Bystrom, S. L. & Graves, M. H. Real-Time Multiplex PCR Assay for Detection of Brucella spp., B. abortus, and B. melitensis. *J Clin Microbiol* **42**, 1290–1293 (2004).
26. Drosten, C. *et al.* Rapid detection and quantification of RNA of Ebola and Marburg viruses, Lassa virus, Crimean-Congo hemorrhagic fever virus, Rift Valley fever virus, dengue virus, and yellow fever virus by real-time reverse transcription-PCR. *J Clin Microbiol* **40**, 2323–2330 (2002).
27. Locatelli, G. *et al.* Real-Time Quantitative PCR for Human Herpesvirus 6 DNA. *J Clin Microbiol* **38**, 4042–4048 (2000).
28. Socolovschi, C., Reynaud, P., Kernif, T., Raoult, D. & Parola, P. Rickettsiae of spotted fever group, Borrelia valaisiana, and Coxiella burnetii in ticks on passerine birds and mammals from the Camargue in the south of France. *Ticks Tick Borne Dis* **3**, 355–360 (2012).
29. Pastorino, B. *et al.* Development of a TaqMan RT-PCR assay without RNA extraction step for the detection and quantification of African Chikungunya viruses. *J Virol Methods* **124**, 65–71 (2005).

30. Ninove, L. *et al.* RNA and DNA bacteriophages as molecular diagnosis controls in clinical virology: a comprehensive study of more than 45,000 routine PCR tests. *PLoS One* **6**, e16142 (2011).
31. Weidmann, M., Meyer-König, U. & Hufert, F. T. Rapid detection of herpes simplex virus and varicella-zoster virus infections by real-time PCR. *J Clin Microbiol* **41**, 1565–1568 (2003).
32. Khanh, T. H. *et al.* Enterovirus 71–associated Hand, Foot, and Mouth Disease, Southern Vietnam, 2011. *Emerg Infect Dis* **18**, 2002–2005 (2012).
33. Benslimani, A., Fenollar, F., Lepidi, H. & Raoult, D. Bacterial Zoonoses and Infective Endocarditis, Algeria. *Emerg Infect Dis* **11**, 216–224 (2005).

Pathogen<br>(no. positive)	Test	Line	Sample	Samples tested<br>No. (%)	Criteria for testing	Positive samples<br>No. (%)
**Confirmed aetiologies**						
N. meningitidis (n=2)	Real-time PCR	P	CSF	257 (100.0)	all	2 (100.0)
	Culture	P	CSF	257 (100.0)	all	1 (50.0)
S. pneumoniae (n=17)	Real-time PCR	P	CSF	257 (100.0)	all	17 (100.0)
	Culture	P	CSF	257 (100.0)	all	6 (35.3)
H. influenzae (n=1)	Real-time PCR	P	CSF	257 (100.0)	all	1 (100.0)
	Culture	P	CSF	257 (100.0)	all	0 (0.0)
L. monocytogenes (n=3)	Real-time PCR	P	CSF	257 (100.0)	all	3 (100.0)
	Culture	P	CSF	257 (100.0)	all	0 (0.0)
Leptospira spp. (n=0)	Real-time PCR	P	CSF	257 (100.0)	all	0 (0.0)
Treponema pallidum (n=1)	Real-time PCR	P	CSF	15 (5.8)	suspicion & randomly	1 (100.0)
	VDRL	P	CSF	232 (90.2)	test availability	1 (100.0)
	Immunoblot	R	CSF	5 (1.9)	PCR positive & suspicion	1 (100.0)
Other bacteria (n=28)	PCR (16S)	R	CSF	140 (54.5)	suspicion & randomly	28 (100.0)
	Sequencing	R	CSF	28 (100.0)	16S positive	28 (100.0)
	Culture	P	CSF	257 (100.0)	all	11 (39.3)
M. tuberculosis (n=34)	Real-time PCR	P	CSF	257 (100.0)	all	20 (58.8)
	Culture	P	CSF	NA	when indicated	2 (5.9)
	Xpert MTB/RIF	R	CSF	20 (7.8)	suspicion	5 (14.7)
	Xpert MTB/RIF Ultra	R	CSF	40 (11.7)	suspicion & negative with other tests	7 (20.6)
Mycobacterium spp. (n=4)	Real-time PCR	R	CSF	48 (18.7)	suspicion	4 (100.0)
Herpes simplex 1&2 (n=2)	Real-time PCR	P	CSF	257 (100.0)	all	2 (100.0)
Varicella-zoster (n=6)	Real-time PCR	P	CSF	257 (100.0)	all	4 (66.7)
	Vesicular eruption	P	NA	257 (100.0)	all	2 (33.3)
	ELISA IgM	P	Serum	16 (6.22)	when indicated	2 (33.3)
Cytomegalovirus (n=1)	Real-time PCR	P	CSF	257 (100.0)	all	1 (100.0)
Enterovirus (n=0)	Real-time PCR	P	CSF	257 (100.0)	all	0 (0.0)
Rabies (n=6)	Real-time PCR	P	CSF & saliva	18 (7.0)	suspicion	6 (100.0)
Epstein-Barr (n=1)	Real-time PCR	P	CSF	52 (20.2)	HIV-positive	1 (100.0)
	Viral capsid antigen IgM/IgG	R	serum	1 (0.4)	Real-time PCR positive	1 (100.0)
Toxoplasma gondii (n=6)	Real-time PCR	P	CSF	54 (21.0)	HIV-positive & suspicion	6 (100.0)
Trypanosoma cruzi (n=5)	Real-time PCR	P	CSF	52 (20.2)	HIV-positive	5 (100.0)
Taenia solium (n=1)	Real-time PCR	P	CSF	52 (20.2)	HIV-positive	0 (0.0)
	ELISA IgG + IB	R	CSF & serum	20 (7.8)	suspicion	1 (100.0)
Cryptococcus spp. (n=20)	India Ink	P	CSF	257 (100.0)	all	14 (70.0)
	Latex	P	CSF	257 (100.0)	all	20 (100.0)
Neurophil antibodies (n=3)	Immunochemistry	R	CSF	54 (21.0)	suspicion & randomly*	3 (100.0)
Anti-NMDA (n=3)	IIFT	R	CSF	3 (1.2)	positive neurophil Ab	3 (100.0)
Respiratory pathogens	Real-time PCR	P & R	NS	200 (77.8)	sample availability	34 (17.0)
**Probable aetiologies**						

Other bacteria (n=13)	CSF characteristics	R	NA	257 (100.0)	all	13 (5.1)
M. tuberculosis (n=3)	Ziehl-Neelsen	P	sputum	NA	when indicated	2 (100.0)
	Xpert MTB/RIF	P	lymph nodes	NA	when indicated	1 (100.0)
Zika virus (n=2)	ELISA IgM	R	Serum	18 (7.9)	suspicion	1 (50.0)
	ELISA IgAM	R	Serum	18 (7.9)	suspicion	1 (50.0)
HIV (n=1)	Immunochemistry	R	CSF	54 (21.0)	randomly	1 (100.0)
JC virus (n=1)	Real-time PCR	R	Serum	28 (10.9)	suspicion	1 (100.0)
Cryptococcus spp. (n=1)	Latex	R	Serum	10 (3.9)	randomly	1 (100.0)
Toxoplasma gondii (n=4)	ELISA IgG	R	Serum	29 (11.3)	suspicion	4 (100.0)
	ELISA IgM, IgA	R	Serum	29 (11.3)	suspicion	0 (100.0)
**Possible aetiologies**						
Influenza B	Real-time PCR	P	NS	200 (77.8)	sample availability	3 (100.0)
Cytomegalovirus	Real-time PCR	P & R	Blood	11 (4.3)	randomly	1 (100.0)
**No diagnosis**						
Leptospira spp.	Real-time PCR	P	CSF	257 (100.0)	all	0 (0.0)
Rickettsia spp.	Real-time PCR	R	CSF	32 (12.4)	randomly	0 (0.0)
Brucella spp.	Real-time PCR	R	CSF	16 (6.2)	suspicion & randomly	0 (0.0)
S. agalactiae	Real-time PCR	P & R	CSF	27 (10.5)	randomly	0 (0.0)
HHV-6	Real-time PCR	P & R	CSF	12 (4.7)	randomly	0 (0.0)
Enterovirus	Real-time PCR	P	CSF	257 (100.0)	all	0 (0.0)
Cytomegalovirus	ELISA IgM	R	Serum	11 (4.3)	randomly	0 (0.0)
Zika virus	Real-time PCR	P	CSF	257 (100.0)	all	0 (0.0)
	Real-time PCR	R	Serum	18 (7.9)	suspicion	0 (0.0)
Dengue virus	Real-time PCR	R	CSF	257 (100.0)	all	0 (0.0)
	Real-time PCR	R	Serum	18 (7.9)	suspicion	0 (0.0)
	ELISA IgM	R	Serum	18 (7.9)	suspicion	0 (0.0)
Mumps	Real-time PCR	P	CSF	257 (100.0)	all	0 (0.0)
JC virus	Real-time PCR	P	CSF	52 (20.2)	HIV-positive	0 (0.0)
EEEV	Real-time PCR	R	CSF	22 (8.6)	randomly	0 (0.0)
St. Louis encephalitis	Real-time PCR	R	CSF	22 (8.6)	randomly	0 (0.0)
WEEV	Real-time PCR	R	CSF	22 (8.6)	randomly	0 (0.0)
VEEV	Real-time PCR	R	CSF	22 (8.6)	randomly	0 (0.0)
Measles	Real-time PCR	R	CSF	4 (1.5)	randomly	0 (0.0)
West Nile	Real-time PCR	R	CSF	15 (5.8)	randomly	0 (0.0)
Chikungunya virus	Real-time PCR	R	Serum	18 (7.9)	suspicion	0 (0.0)
	ELISA IgM	R	Serum	18 (7.9)	suspicion	0 (0.0)
Yellow fever	Real-time PCR	R	Serum	15 (5.8)	randomly	0 (0.0)
Bartonella spp.	Real-time PCR	R	Blood	21 (8.2)	randomly	0 (0.0)
Epstein-Barr	Real-time PCR	R	Blood	11 (4.3)	HIV-positive	0 (0.0)
Toxoplasma gondii	Real-time PCR	R	Blood	29 (11.3)	HIV-positive	0 (0.0)

*3 with a specific clinical suspicion, 22 with no diagnostic, 29 with a confirmed infectious aetiology

Abbreviations: WEEV, Western Equine Encephalitis; VEEV, Venezuelan Equine Encephalitis; EEEV, Eastern Equine Encephalitis; P, prospective; R, retrospective; IB, Immunoblot; PCR, polymerase chain reaction; ab, antibodies; CSF, cerebrospinal fluid; IIFT, Indirect immunofluorescent test; ELISA, Enzyme-linked immunosorbent assay; Anti-NMDA, N-methyl-D-aspartate receptor; HIV, human immunodeficiency virus; NS, nasopharyngeal swab; NA, no data available.

Supplementary Table S4. Number of samples tested and aetiologies identified by diagnostic method in patients with suspected central nervous system. Bolivia, 2017-2018

	Confirmed	Probable	Possible
**Bacteria**			
Streptococcus pneumoniae<br>*Neisseria meningitidis*<br>*Haemophilus influenzae*<br>*Listeria monocytogenes*	DNA detected by qPCR or isolation from CSF	NA	NA
Treponema pallidum	DNA detected by qPCR in CSF; CSF VDRL reactive (Wiener lab. Santa Fe, Argentina) [a] or immunoblot (Euroimmun, Lübeck, Germany) positive	NA	NA
Other Gram-positive or Gram-negative bacteria[b]	Isolation from CSF and/or 16S rRNA gene amplification by PCR and sequencing	CSF and blood procalcitonin concentration >0.5 ng/ml (B-R-A-H-M-S, Thermo Fisher Scientific, Waltham, USA) [a], AND/OR CSF examination showing at least one of the following characteristics: Turbid appearance, leukocytosis (>100 cell/mm^3) or Leukocytosis (10-100 cell/mm^3) AND either an elevated protein (>100mg/dl) or decreased glucose (<40mg/dl) (WHO criteria)	NA
**Mycobacteria**			
M. tuberculosis	Isolation from CSF (Lowenstein-Jensen medium)[a]; OR<br>DNA detected by qPCR; Xpert MTB/RIF or Ultra Xpert MTB/RIF (Cepheid, California, USA) from CSF	Acid-fast bacilli seen in the sputum by Ziehl-Neelsen stain[a]; OR<br>Isolation (Lowenstein-Jensen medium) or DNA detection by qPCR in a sample other than CSF[a]	NA
Mycobacterium spp.	DNA detected by qPCR from CSF	NA	NA
**Viruses**		NA	NA
Herpes simplex virus 1&2	DNA detected by qPCR in CSF	NA	NA
Human cytomegalovirus	DNA detected by qPCR in CSF	NA	DNA detected by qPCR in blood
Varicella-zoster virus	DNA detected by qPCR in CSF, OR<br>typical vesicular eruption AND ELISA IgM positive (Liaison. Diasorin, Italy)	NA	NA
Rabies virus	RNA detected by qPCR in CSF and saliva	NA	NA
Epstein-Barr	DNA detected by qPCR in CSF AND IgM/IgG viral capsid antigen positive (Liaison. Diasorin, Italy)	NA	NA
JC virus	NA	Intrathecal IgG by IgG quantification in serum and CSF using Optilite (The Binding Site Group, Birmingham, UK), JC virus in serum by qPCR and compatible imaging features	NA

Zika virus	NA	Detection of Zika IgM or IgAM antibodies in serum by ELISA (Euroimmun. Lübeck, Germany) in serum	NA
Human immunodeficiency virus (HIV)	NA	GFAP expression by immunohistochemistry in CSF	NA
Influenza virus B	NA	NA	RNA detected in nasopharyngeal swab by qPCR AND no other cause found
**Fungi**			
Cryptococcus spp.	Positive India ink from CSF[a]; OR<br>Antigen detected by Cryptococcal Latex Agglutination System (CALAS, Meridian Bioscience, Inc., Cincinnati, USA) in CSF	Antigen detected by Cryptococcal Latex Agglutination System in Blood (CALAS, Meridian. Diagnostics, Cincinnati, USA)	NA
**Parasites**			
Toxoplasma gondii	DNA detected by qPCR in CSF	High *T. gondii* specific IgG antibodies (>800 UI/ml) by ELISA (VIDAS, bioMérieux. Marcy-l'Étoile, France) in serum with imaging features compatible with *T. gondii*, and already in a treatment	NA
Taenia solium	Specific IgG antibodies in serum and CSF by ELISA (IBL international. Hamburg, Germany) and confirmed by EITB (LDBIO. Lyon, France)	NA	NA
Trypanosoma cruzi	DNA detected by qPCR in CSF	NA	NA
**Autoimmune**			
Anti-NMDAR	Detection of neurophil antibodies in the CSF by immunohistochemistry AND confirmed by indirect immunofluorescence test (Euroimmun. Lübeck, Germany)	NA	NA

[a]Results obtained from the hospital medical record

[b]Other Gram-positive or Gram-negative bacteria: *A. baumannii, Acinetobacter* spp¸ *Achromobacter* spp., *E. coli, E. faecium, Burkholderia* spp., *K. pneumoniae, P. aeruginosa, Pseudomona* spp., *S. aureus, Staphylococcus* spp., *S. epidermidis S. maltophilia, S. pyogenes*¸ *Streptococcus spp*.

Abbreviations: NA, No cases met during the current study; DNA, deoxyribonucleic acid; qPCR, real-time polymerase chain reaction; RNA, ribonucleic Acid; rRNA, ribosomal ribonucleic acid; CSF, cerebrospinal fluid; VDRL, venereal disease research laboratory test; WHO, World Health Organization; EITB, enzyme-linked immunoelectrotransfer blot; ELISA, enzyme-linked immunosorbent assay; GFAP, glial fibrillary acidic protein; NMDAR, *N*-Methyl-D-aspartic acid receptor

Supplementary Table S5. Laboratory tests and other evidence used to define cases as confirmed, probable or possible aetiologies in patients with suspected central nervous system infections. Bolivia, 2017-2018

Aetiology	All<br><br>no.	Fever<br><br>no. (%)	Nausea and/or Vomiting<br><br>no. (%)	Neck stiffness or bulging fontanelle<br><br>no. (%)	Headache (>2.5 yo)<br><br>no. (%)	Seizures<br><br>no. (%)	GCS <15<br><br>no. (%)	GCS<br><br>Median (range)	Cough<br><br>no. (%)	Rash<br><br>no. (%)	Aphasia, paresis or vision loss<br><br>no. (%)
**Bacteria**	46	44 (95.7)	27 (58.7)	30 (65.2)	26/32 (81.3)	14 (30.4)	29/38 (76.3)	12 (3-15)	14 (30.4)	5 (10.9)	10 (21.7)
S. pneumoniae	15	15 (100.0)	11 (73.3)	15 (100.0)	12/13 (92.3)	3 (20.0)	10/14 (71.4)	14 (4-15)	7 (46.7)	2 (13.3)	4 (26.7)
N. meningitidis	2	1 (100.0)	2 (100.0)	1 (50.0)	1 (50.0)	0 (0.0)	2 (100.0)	12.5 (11-14)	1 (50.0)	0 (0.0)	1 (50.0)
H. influenzae type b	1	1 (100.0)	1 (100.0)	0 (0.0)	NA[§]	1 (100)	NA[¶]	NA[¶]	1 (50.0)	0 (0.0)	0 (0.0)
L. monocytogenes	3	2 (66.7)	0 (0.0)	2 (66.7)	2 (66.7)	0 (0.0)	3 (100.0)	10 (6-10)	0 (0.0)	0 (0.0)	3 (100.0)
T. pallidum	1	1 (100.0)	1 (100.0)	1 (100.0)	NA[§]	0 (0.0)	NA[¶]	NA[¶]	0 (0.0)	0 (0.0)	0 (0.0)
Other bacteria[*]	24	24 (100.0)	12 (50.0)	11 (45.8)	11/15 (73.3)	10 (41.7)	14/19 (73.7)	12 (3-15)	5 (20.8)	3 (12.5)	2 (8.3)
Mycobacteria	33	33 (100.0)	20 (60.6)	23 (69.7)	19 (57.6)	11 (33.3)	27 (81.8)	11 (4-15)	17 (51.5)	1 (3.0)	3 (9.1)
M. tuberculosis	29	29 (100.0)	16 (55.2)	20 (69.0)	15 (51.7)	9 (31.0)	23/26 (88.5)	11 (4-15)	14 (48.3)	0 (0.0)	2 (6.9)
Mycobacterium spp.	4	4 (100.0)	4 (100.0)	3 (75.0)	4 (100.0)	2 (50.0)	4 (100.0)	11 (9-14)	3 (75.0)	1 (25.0)	1 (25.0)
**Viruses**	14	11 (78.6)	8 (57.1)	6 (42.9)	6 (42.9)	6 (42.9)	9/13 (69.2)	3 (4-15)	4 (28.6)	4 (28.6)	4 (28.6)
Rabies[†]	6	6 (100.0)	4 (66.7)	2 (33.3)	2 (33.3)	4 (66.7)	4/5 (80.0)	13 (13-15)	1 (16.7)	0 (0.0)	3 (50.0)
Varicella-zoster virus	6	3 (50.0)	4 (66.7)	2 (33.3)	2 (33.3)	2 (33.3)	4 (66.7)	8.5 (4-15)	1 (16.7)	4 (66.7)	1 (16.7)
Herpes simplex 1	1	1 (100.0)	1 (100.0)	1 (100.0)	1 (100.0)	0 (0.0)	1 (100.0)	11	0 (0.0)	0 (0.0)	0 (0.0)
Herpes simplex 2	1	1 (100.0)	1 (100.0)	1 (100.0)	1 (100.0)	0 (0.0)	0 (0.0)	15	1 (0.0)	0 (0.0)	0 (0.0)
**Fungi**	16	16 (100.0)	14 (87.5)	9 (56.2)	15 (93.8)	6 (37.5)	7 (43.8)	15 (9-15)	5 (31.2)	4 (25.0)	3 (18.7)
C.neoformans	10	10 (100.0)	10 (100.0)	5 (50.0)	10 (100.0)	5 (50.0)	5 (50.0)	14.5 (11-15)	3 (60.0)	2 (20.0)	3 (30.0)
Cryptococcus spp.	6	6 (100.0)	4 (66.7)	4 (66.7)	5 (83.3)	1 (16.7)	2 (33.3)	15 (9-15)	2 (33.3)	2 (33.3)	0 (0.0)
**Parasites**	9	5 (55.6)	4 (44.4)	3 (33.3)	3 (33.3)	7 (77.8)	4/8 (50.0)	14.5 (6-15)	3 (33.3)	0 (0.0)	7 (77.8)
Toxoplasma gondii	5	3 (60.0)	2 (40.0)	2 (40.0)	2 (40.0)	4 (80.0)	3 (60.0)	14 (6-15)	2 (40.0)	0 (0.0)	4 (80.0)
Trypanosoma cruzi	3	2 (66.7)	2 (66.7)	1 (33.3)	1 (33.3)	2 (66.7)	1 (33.3)	15 (11-15)	1 (33.3)	0 (0.0)	3 (100.0)
Taenia solium	1	0 (0.0)	0 (0.0)	0 (0.0)	0 (0.0)	1 (100.0)	NA[¶]	NA[¶]	0 (0.0)	0 (0.0)	0 (0.0)
**Co-infections**	10	8 (80.0)	5 (50.0)	7 (70.0)	7/9 (77.8)	2 (20.0)	7/8 (87.5)	11 (4-15)	5 (50.0)	0 (0.0)	1 (10.0)

S. pneumoniae + cytomegalovirus	1	1 (100.0)	1 (100.0)	0 (0.0)	NA[§]	1 (100.0)	NA[¶]	NA[¶]	1 (100.0)	0 (0.0)	0 (0.0)
S. pneumoniae + *Cryptococcus* spp.	1	1 (100.0)	1 (100.0)	0 (0.0)	1 (100.0)	0 (0.0)	0 (0.0)	15	0 (0.0)	0 (0.0)	0 (0.0)
M. tuberculosis + *C. neoformans*	1	1 (100.0)	0 (0.0)	1 (100.0)	1 (100.0)	0 (0.0)	1 (100.0)	9	1 (100.0)	0 (0.0)	0 (0.0)
M. tuberculosis + other bacteria[‡]	4	4 (100.0)	1 (25.0)	3 (75.0)	2 (50.0)	0 (0.0)	3/4 (75.0)	7 (4-14)	1 (25.0)	0 (0.0)	1 (25.0)
C. neoformans + *T. cruzi*	2	0 (0.0)	1 (50.0)	2 (100.0)	2 (100.0)	0 (0.0)	2 (100.0)	13.5 (13-14)	1 (50.0)	0 (0.0)	0 (0.0)
T. gondii + Epstein-Barr	1	1 (100.0)	1 (100.0)	1 (100.0)	1 (100.0)	1 (100.0)	1 (100.0)	7	1 (100.0)	0 (0.0)	0 (0.0)
**Autoimmune**<br>**Anti-NMDAR**	3	3 (100.0)	2 (66.7)	0 (0.0)	2/2 (100.0)	3 (100.0)	2/2 (100.0)	10.5 (10-11)	1 (33.3)	1 (33.3)	0 (0.0)

[*]*K. pneumoniae* (n=6), *A. baumannii* (n=4), *P. aeruginosa* (n=2), *S. aureus* (n=2), *Streptococcus* spp. (n=2), *E. coli* (n=2), *S. pyogenes* (n=1), *E. faecium* (n=1) *S. epidermidis* (n=1), *Achromobacter* spp. (n=1), *S. maltophilia* (n=1), *Staphylococcus* spp. (n=1)

[†]Specific symptoms: Hydrophobia, aerophobia, and photophobia

[‡]*Acinetobacter* spp. (n=1), *Burkholderia* spp. (n=1), *Pseudomona* spp. (n=1), *S. epidermidis* (n=1)

[§]Patients <2.5 years old

[¶]Not testable or no data available

Abbreviations: GCS= Glasgow Coma Scale, Hb= Hemoglobin, NMDAR=N-Methyl-D aspartic acid receptor; yo, years old.

Supplementary Table S6. Clinical features of patients with suspected central nervous system infection by confirmed aetiologies. Bolivia, 2017-2018

	Hemoglobin <12g/dL no. (%)	Leukocytosis >10,000/mm3 no. (%)	Leukocytes Median (range)	Neutrophilia >65% no. (%)	Thrombocytopenia <150,000 cell/mm^3 no. (%)	Glycemia ≥130 mg/dL no. (%)	Hyponatremia <145 mEq/L no. (%)	Hypokalemia <3.5 mEq/L no. (%)	Urea >45 mg/dL no. (%)	Creatinine >1.3 mg/dL no. (%)
**Bacteria (n=46)**	30 (65.2)	35 (76.0)	14,795 (2,800-31,300)	37 (80.4)	11/45 (24.4)	25/40 (62.5)	15/42 (35.7)	6/38 (15.8)	12/42 (28.6)	6/42 (14.3)
Streptococcus pneumoniae (n=15)	8 (53.3)	12 (80.0)	17,900 (2,800-31,300)	13 (86.7)	2 (13.3)	10 (66.7)	3/14 (21.4)	2/14 (14.3)	4/14 (28.6)	2 (13.3)
Neisseria meningitidis (n=2)	2 (100.0)	2 (100.0)	17,350 (15,400-19,300)	2 (100.0)	1 (50.0)	1 (50.0)	0 (0.0)	0 (0.0)	1 (50.0)	0 (0.0)
Haemophilus influenzae type b (n=1)	1 (100.0)	0 (0.0)	4,900	0 (0.0)	0 (0.0)	0 (0.0)	1 (100.0)	NA	1 (50.0)	0 (0.0)
Listeria monocytogenes (n=3)	1 (33.3)	0 (0.0)	8,100 (6,800-8,100)	3 (100.0)	1 (33.3)	2 (66.7)	1 (33.3)	0 (0.0)	1 (33.3)	0 (0.0)
Treponema pallidum (n=1)	1 (100.0)	1 (100.0)	12,700	1 (100.0)	1 (100.0)	0 (0.0)	1 (100.0)	0 (0.0)	NA	NA
Other bacteria[*] (n=24)	17 (70.8)	20 (83.3)	14,200 (4,700-27,740)	18 (75.0)	6/23 (26.1)	12/18 (66.7)	9/22 (40.9)	4/18 (22.2)	5/22 (22.7)	4/21 (19.0)
***Mycobacteria* (n=33)**	13 (39.4)	13 (39.4)	9,100 (4,500-23,500)	33 (100.0)	3 (9.1)	10/27 (37.0)	18/32 (56.2)	6/31 (19.3)	4/32 (12.5)	4/32 (12.5)
Mycobacterium tuberculosis (n=29)	12 (41.4)	11 (37.9)	9,100 (4,500-23,500)	29 (100.0)	3 (10.3)	8/25 (32.0)	16/28 (57.1)	5/27 (18.5)	3/28 (10.7)	3/28 (10.7)
Mycobacterium spp. (n=4)	1 (25.0)	2 (50.0)	9,600 (7,300-13,380)	4 (100.0)	0 (0.0)	2 (50.0)	2 (50.0)	1 (25.0)	1 (25.0)	0 (0.0)
**Viruses (n=14)**	6 (42.9)	6 (42.9)	9,700 (6,600-14,200)	10 (71.4)	2 (14.3)	3/11 (27.3)	5 (35.7)	1 (7.1)	5/13 (38.5)	1/12 (8.3)
Rabies (n=6)	2 (33.3)	4 (66.7)	7,500 (7,500-14,200)	5 (83.3)	0 (0.0)	1/4 (25.0)	3 (50.0)	1 (16.7)	3 (50.0)	0/4 (0.0)
Varicella-zoster virus (n=6)	4 (66.7)	1 (16.7)	8,610 (6,600-10,390)	3 (50.0)	2 (33.3)	1/5 (20.0)	2 (33.3)	0 (0.0)	2 (33.3)	1 (16.7)
Herpes simplex 1 (n=1)	0 (0.0)	0 (0.0)	9,700	1 (100.0)	0 (0.0)	0 (0.0)	0 (0.0)	0 (0.0)	0 (0.0)	0 (0.0)
Herpes simplex 2 (n=1)	0 (0.0)	1 (100.0)	10,600	1 (100.0)	0 (0.0)	1 (50.0)	0 (0.0)	0 (0.0)	NA	0 (0.0)
**Fungi n=16**	10 (62.5)	1 (6.2)	6,320 (2,340-13,800)	16 (100.0)	4 (25.0)	2/15 (13.3)	6/15 (40.0)	7/15 (46.7)	2 (12.5)	1 (6.25)
Cryptococcus neoformans (n=10)	7 (70.0)	1 (10.0)	6,320 (2,340-13,800)	10 (100.0)	3 (30.0)	1/9 (11.1)	6/9 (66.7)	4/8 (50.0)	1 (10.0)	0 (0.0)
Cryptococcus spp. (n=6)	3 (50.0)	0 (0.0)	6,350 (2,700-9,400)	6 (100.0)	1 (16.7)	1 (16.7)	0 (0.0)	3 (50.0)	1 (16.7)	1 (16.7)
**Parasites (n=9)**	8 (88.9)	0 (0.0)	5,700 (1,800-9,500)	6 (66.7)	1 (11.1)	2 (22.2)	4 (44.4)	1 (11.1)	5 (55.6)	2 (22.2)
Toxoplasma gondii (n=5)	5 (100.0)	0 (0.0)	4,680 (1,800-9,500)	4 (80.0)	0 (0.0)	1 (20.0)	2 (40.0)	1 (20.0)	2 (40.0)	0 (0.0)
Trypanosoma cruzi (n=3)	2 (66.7)	0 (0.0)	5,700 (5,700-7,600)	1 (33.3)	1 (33.3)	0 (0.0)	1 (33.3)	0 (0.0)	2 (66.7)	2 (66.7)
Taenia solium (n=1)	1 (100.0)	0 (0.0)	6,200	1 (100.0)	0 (0.0)	1 (100.0)	1 (100.0)	0 (0.0)	1 (100.0)	0 (0.0)
**Co-infections (n=10)**	5 (40.0)	4 (40.0)	9,195 (2,960-19,500)	8 (80.0)	1 (10.0)	4/9 (44.4)	5 (50.0)	3 (30.0)	3 (30.0)	1/9 (11.1)
S. pneumoniae + cytomegalovirus (n=1)	1 (100.0)	1 (100.0)	15,500	0 (0.0)	0 (0.0)	0 (0.0)	0 (0.0)	0 (0.0)	0 (0.0)	NA
S. pneumoniae + *Cryptococcus* spp. (n=1)	1 (100.0)	0 (0.0)	7,230	1 (100.0)	0 (0.0)	0 (0.0)	0 (0.0)	0 (0.0)	1 (100.0)	1 (100.0)
M. tuberculosis + *C. neoformans* (n=1)	1 (100.0)	1 (100.0)	10,100	1 (100.0)	0 (0.0)	1 (100.0)	1 (100.0)	1 (100.0)	0 (0.0)	0 (0.0)
M. tuberculosis + other bacteria[†] (n=4)	1 (25.0)	2 (50.0)	12,260 (2,960-19,500)	4 (100.0)	1 (25.0)	2/3 (66.7)	3 (75.0)	1 (25.0)	1 (25.0)	0 (0.0)
C. neoformans + *T. cruzi* (n=2)	1 (50.0)	0 (0.0)	4,880 (4,500-5,260)	1 (50.0)	0 (0.0)	1 (50.0)	0 (0.0)	1 (50.0)	0 (0.0)	0 (0.0)
T. gondii + Epstein-Barr (n=1)	0 (0.0)	0 (0.0)	8,400	1 (100.0)	0 (0.0)	0 (0.0)	1 (100.0)	0 (0.0)	1 (100.0)	0 (0.0)
**Autoimmune (Anti-NMDAR) (n=3)**	2 (66.6)	0 (0.0)	6,940 (4,300-7,000)	1 (33.3)	1 (33.3)	0 (0.0)	1 (33.3)	0 (0.0)	0/2 (0.0)	0 (0.0)

K. pneumoniae (n=6), *A. baumannii* (n=4), *P. aeruginosa* (n=2), *S. aureus* (n=2), *Streptococcus* spp. (n=2), *E. coli* (n=2), *S. pyogenes* (n=1), *E. faecium* (n=1) *S. epidermidis* (n=1), *Achromobacter* spp. (n=1), *S. maltophilia* (n=1), *Staphylococcus* spp. (n=1). † *Acinetobacter* spp. (n=1), *Burkholderia* spp. (n=1), *Pseudomona* spp. (n=1), *S. epidermidis* (n=1)

Abbreviations: AST, Aspartate transaminase; Anti-NMDAR, N-Methyl-D-aspartic acid receptor; NA, no data available.

Suppplementary Table S7. Blood parameters of patients with suspected central nervous system infection by confirmed aetiologies. Bolivia, 2017-2018

	Appearance					White blood cell count /mm³	Neutrophils (%)	Proteins (mg/dL)	CSF/serum glucose (%)
	**Clear no. (%)**	**Slightly opal. no. (%)**	**Opal. + xant. no. (%)**	**xant. no. (%)**	**Opal. or turbid no. (%)**	**Median (range)**	**no (%)**	**Median (range)**	**Median (range)**
**Bacteria (n=46)**	3 (6.5)	2 (4.4)	7 (15.2)	0 (0.0)	34 (73.9)	236 (3-27,100)	32 (69.6)	227 (10-816)	7.2 (0-65)
Streptococcus pneumoniae (n=15)	0 (0.0)	0 (0.0)	0 (0.0)	0 (0.0)	15 (100.0)	300 (3-20,110)	10 (66.7)	285 (10-816)	5 (1.2-39.8)
Neisseria meningitidis (n=2)	0 (0.0)	0 (0.0)	0 (0.0)	0 (0.0)	2 (100.0)	13,280 (360-26,200)	2 (100.0)	258.5 (200-317)	6.6 (6.6)
Haemophilus influenzae type b (n=1)	0 (0.0)	0 (0.0)	0 (0.0)	0 (0.0)	1 (100.0)	980	1 (100.0)	297	9.3
Listeria monocytogenes (n=3)	0 (0.0)	0 (0.0)	2 (66.7)	0 (0.0)	1 (33.3)	77 (2-200)	1 (33.3)	83 (52-122)	17.1 (6-24.5)
Treponema pallidum (n=1)	1 (100)	0 (0.0)	0 (0.0)	0 (0.0)	0 (0.0)	4	0 (100.0)	106	72
Other bacteria* (n=24)	2 (8.3)	2 (8.3)	5 (20.9)	0 (0.0)	15 (62.5)	229.5 (3-27,100)	18 (75.0)	235 (38-810)	11.1 (0-65)
***Mycobacteria* (n=33)**	0 (0.0)	0 (0.0)	0 (0.0)	33 (100.0)	0 (0.0)	160 (1-1,277)	3 (9.0)	150 (10-932)	20.5 (4.3-72)
Mycobacterium tuberculosis (n=29)	0 (0.0)	0 (0.0)	0 (0.0)	29 (100.0)	0 (0.0)	150 (1-1,277)	2 (6.9)	140 (10-932)	20.4 (4.3-72)
Mycobacterium spp. (n=4)	0 (0.0)	0 (0.0)	0 (0.0)	4 (100.0)	0 (0.0)	190 (22-200)	1 (25.0)	164 (70-270)	21.7 (19.5-44.6)
**Viruses (n=14)**	13 (92.9)	1 (7.1)	0 (0.0)	0 (0.0)	0 (0.0)	5 (1-100)	0 (0.0)	45 (20-150)	58 (45-80)
Rabies (n=6)	6 (100.0)	0 (0.0)	0 (0.0)	0 (0.0)	0 (0.0)	1 (1-11)	0 (0.0)	34 (20-58)	62.5 (45-72.6)
Varicella-zoster virus (n=6)	6 (100.0)	0 (0.0)	0 (0.0)	0 (0.0)	0 (0.0)	4 (0-40)	0 (0.0)	27.7 (12-150)	57.9 (56.6-80)
Herpes simplex 1 (n=1)	1 (100.0)	0 (0.0)	0 (0.0)	0 (0.0)	0 (0.0)	5	0 (0.0)	23	47.7
Herpes simplex 2 (n=1)	0 (0.0)	1 (100.0)	0 (0.0)	0 (0.0)	0 (0.0)	100	0 (0.0)	88	48.6
**Fungi n=16**	5 (31.3)	8 (50.0)	0 (0.0)	3 (18.7)	0 (0.0)	20 (3-360)	1 (6.3)	45 (1-216)	28 (4.9-38)
Cryptococcus neoformans (n=10)	3 (30.0)	6 (60.0)	0 (0.0)	1 (10.0)	0 (0.0)	20 (3-300)	0 (0.0)	45 (1-216)	24.5 (4.9-38)
Cryptococcus spp. (n=6)	2 (33.3)	2 (33.3)	0 (0.0)	2 (33.3)	0 (0.0)	21.5 (5-360)	1 (100.0)	45 (15-114)	33 (27.4-37)
**Parasites (n=9)**	8 (88.9)	1 (11.1)	0 (0.0)	0 (0.0)	0 (0.0)	5 (2-100)	0 (0.0)	58 (10-112)	46 (31-66)
Toxoplasma gondii (n=5)	5 (100.0)	0 (0.0)	0 (0.0)	0 (0.0)	0 (0.0)	5 (2-100)	0 (0.0)	88 (68-112)	46.7 (42.5-53.2)
Trypanosoma cruzi (n=3)	2 (66.7)	1 (33.3)	0 (0.0)	0 (0.0)	0 (0.0)	5 (5-50)	0 (0.0)	29 (10-48)	37.4 (31.8-37.4)
Taenia solium (n=1)	1 (100.0)	0 (0.0)	0 (0.0)	0 (0.0)	0 (0.0)	5	0 (0.0)	NA	55

**Co-infections (n=10)**	2 (20.0)	0 (0.0)	1 (10.0)	6 (60.0)	1 (10.0)	101 (50-1,868)	1 (10.0)	205 (10-639)	17 (9.7-40.6)
S. pneumoniae + cytomegalovirus (n=1)	0 (0.0)	0 (0.0)	0 (0.0)	0 (0.0)	1 (100.0)	1868	0 (0.0)	639	7.3
S. pneumoniae + *Cryptococcus* spp. (n=1)	0 (0.0)	0 (0.0)	0 (0.0)	1 (100.0)	0 (0.0)	50	0 (0.0)	10	17
M. tuberculosis + *C. neoformans* (n=1)	0 (0.0)	0 (0.0)	0 (0.0)	1 (100.0)	0 (0.0)	50	0 (0.0)	270	16.4
M. tuberculosis + other bacteria[†] (n=4)	0 (0.0)	0 (0.0)	1 (25.0)	3 (75.0)	0 (0.0)	176 (100-300)	1 (25.0)	287.5 (110-560)	10.9 (9.7-19.5)
C. neoformans + *T. cruzi* (n=2)	1 (50.0)	0 (0.0)	0 (0.0)	1 (50.0)	0 (0.0)	127.5 (5-250)	0 (0.0)	39 (32-46)	26.3 (18.5-34)
Toxoplasma gondii + Epstein-Barr (n=1)	1 (100.0)	0 (0.0)	0 (0.0)	0 (0.0)	0 (0.0)	80	0 (0.0)	180	40.6
**Autoimmune (Anti-NMDAR) (n=3)**	3 (100.0)	0 (0.0)	0 (0.0)	0 (0.0)	0 (0.0)	2 (2-150)	0 (0.0)	25 (12-60)	73.8 (63-75.3)
**Non-infectious (n=52)**	43 (82.7)	1 (1.9)	0 (0.0)	8 (15.4)	0 (0.0)	5 (0-50)	0 (0.0)	32.5 (7-180)	52 (36-278)
**Unknown (n=46)**	40 (87.0)	1 (2.2)	0 (0.0)	5 (10.8)	0 (0.0)	5 (0-4350)	0 (0.0)	45.5 (1-535)	47 (7-300)

[*]*K. pneumoniae* (n=6), *A. baumannii* (n=4), *P. aeruginosa* (n=2), *S. aureus* (n=2), *Streptococcus spp*. (n=2), *E. coli* (n=2), *S. pyogenes* (n=1), *E. faecium* (n=1) *S. epidermidis* (n=1), *Achromobacter* spp. (n=1), *S. maltophilia* (n=1), *Staphylococcus* spp. (n=1). [†]*Acinetobacter* spp. (n=1), *Burkholderia* spp. (n=1), *Pseudomona* spp. (n=1), *S. epidermidis* (n=1)
Abbreviations: Anti-NMDAR, *N*-Methyl-D-aspartic acid receptor; xant, xanthochromia; opal, opalescent; CSF, cerebrospinal fluid; NA, no data available.

Supplementary Table S8. Cerebrospinal fluid parameters of patients with suspected central nervous system infection by confirmed aetiologies. Bolivia, 2017-2018

	Respiratory pathogens												
**CNS aetiologies (no. tested)**	**HRV no. (%)**	**VRS A/B no. (%)**	**CoV-HKU1 no. (%)**	**Flu B no. (%)**	**CoV-43 no. (%)**	**MPV no. (%)**	**Flu AH1N1 no. (%)**	**CoV-63 no. (%)**	**HBoV no. (%)**	**ADV no. (%)**	**Co-infection no. (%) or pathogens**	**Negative no. (%)**	**Total no. (%)**
**Confirmed infectious (n=94)**	**6 (6.4)**	**2 (66.7)**	**1 (66.7)**	**1 (66.7)**	**2 (100.0)**	**2 (100.0)**	**1 (100.0)**	**1 (100.0)**	**0 (0.0)**	**1 (100.0)**	**2 (40.0)**	**75 (79.8)**	**19 (20.2)**
Cryptococcus spp. (n=10)	2 (20)	1 (10.0)	0 (0.0)	0 (0.0)	1 (10.0)	1 (10.0)	0 (0.0)	0 (0.0)	0 (0.0)	0 (0.0)	HRV + HBoV	4 (40.0)	6 (60.0)
Toxoplasma gondii (n=5)	0 (0.0)	1 (20)	0 (0.0)	1 (20)	0 (0.0)	0 (0.0)	0 (0.0)	0 (0.0)	0 (0.0)	0 (0.0)	0 (0.0)	3 (60.0)	2 (40.0)
Mycobacterium spp. (n=27)	1 (3.7)	0 (0.0)	0 (0.0)	0 (0.0)	0 (0.0)	1 (3.7)	0 (0.0)	0 (0.0)	0 (0.0)	1 (3.7)	0 (0.0)	25 (92.6)	2 (7.4)
Varicella-zoster (n=6)	2 (33.3)	0 (0.0)	0 (0.0)	0 (0.0)	0 (0.0)	0 (0.0)	0 (0.0)	0 (0.0)	0 (0.0)	0 (0.0)	0 (0.0)	4 (66.7)	2 (33.3)
Streptococcus pneumoniae (n=10)	0 (0.0)	0 (0.0)	0 (0.0)	0 (0.0)	1 (10.0)	0 (0.0)	0 (0.0)	0 (0.0)	0 (0.0)	0 (0.0)	0 (0.0)	9 (90.0)	1 (10.0)
Listeria monocytogenes (n=3)	0 (0.0)	0 (0.0)	0 (0.0)	0 (0.0)	0 (0.0)	0 (0.0)	0 (0.0)	1 (33.3)	0 (0.0)	0 (0.0)	0 (0.0)	2 (66.7)	1 (33.3)
Haemophilus influenzae (n=1)	0 (0.0)	0 (0.0)	1 (100)	0 (0.0)	0 (0.0)	0 (0.0)	0 (0.0)	0 (0.0)	0 (0.0)	0 (0.0)	0 (0.0)	0 (0.0)	1 (100.0)
Other bacteria* (n=14)	1 (7.1)	0 (0.0)	0 (0.0)	0 (0.0)	0 (0.0)	0 (0.0)	1 (7.1)	0 (0.0)	0 (0.0)	0 (0.0)	HRV + HEV	11 (78.6)	3 (21.4)
**Probable infectious (n=18)**	0 (0.0)	0 (0.0)	0 (0.0)	0 (0.0)	0 (0.0)	0 (0.0)	0 (0.0)	0 (0.0)	0 (0.0)	0 (0.0)	0 (0.0)	18 (100)	0 (0.0)
**Possible infectious (n=4)**	0 (0.0)	0 (0.0)	0 (0.0)	2 (50.0)	0 (0.0)	0 (0.0)	0 (0.0)	0 (0.0)	0 (0.0)	0 (0.0)	HRV + Flu B	1 (25)	3 (75.0)
**Non-infectious (n=45)**	4 (8.9)	1 (2.2)	1 (2.2)	0 (0.0)	0 (0.0)	0 (0.0)	0 (0.0)	0 (0.0)	1 (2.2)	0 (0.0)	0 (0.0)	38 (84.4)	7 (15.6)
**Unknown (n=39)**	2 (5.1)	0 (0.0)	1 (2.6)	0 (0.0)	0 (0.0)	0 (0.0)	0 (0.0)	0 (0.0)	0 (0.0)	0 (0.0)	CoV-NL63+ HEV<br>MPV + HIPV3	34 (87.2)	5 (12.8)
Total Respiratory pathogens (n=200)	**12 (6.0)**	**3 (1.5)**	**3 (1.5)**	**3 (1.5)**	**2 (1.0)**	**2 (1.0)**	**1 (0.5)**	**1 (0.5)**	**1 (0.5)**	**1 (0.5)**	**5 (2.5)**	**166 (83.0)**	**34 (17.0)**

S. aureus, Achromobacter spp. *Staphylococcus* spp.
Abbreviations: CNS, Central nervous system; HRV, human rhinovirus; VRS A/B, Respiratory syncytial virus; CoV, Coronavirus; Flu, influenza; MPV, human metapneumovirus; HBoV, Human bocavirus; ADV, adenovirus; HEV, human enterovirus; HIPV3, Human parainfluenza virus 3

Supplementary Table S9. Respiratory pathogens in patients with suspected central nervous system infection by aetiologies. Bolivia, 2017-2018

	Patients<br><br>no.	Male<br><br>no.	Female<br><br>no.	Children <15 yo<br><br>no.	Adult ≥15 yo<br><br>no.	HIV-positive<br><br>no.	HIV-negative<br><br>no.	Fever >37.5°C<br><br>no.	Focal lesion or abscess<br><br>no.
N. meningitidis	1	1	0	0	1	0	1	0	0
Varicella-zoster	4	2	2	2	2	0	4	1	0
Trypanosoma cruzi	1	1	0	0	1	1	0	0	1
C. neoformans + T. cruzi	2	1	1	0	2	2	0	0	1
Toxoplasma gondii	3	2	1	0	3	3	0	1	3
S. pneumoniae + *Cryptococcus* spp.	1	1	0	0	1	0	1	1	0
Cryptococcus spp.	2	2	0	0	2	1	1	2	NA
L. monocytogenes	1	1	0	0	1	1	0	0	NA
Taenia solium	1	1	0	0	1	1	0	0	1
**Total no. (%)**	**16 (100.0)**	**12 (75.0)**	**4 (25.0)**	**2 (12.5)**	**14 (87.5)**	**9 (56.2)**	**7 (43.8)**	**5 (31.2)**	**6 (37.5)**
Abbreviations: NA, no data available									

Supplementary Table S10. Confirmed infectious aetiologies that did not fulfil the modified World Health Organization (WHO) criteria for central nervous system infection. Bolivia, 2017-2018

	**Died n=94**<br><br>**no.**	**Survived n=129**<br><br>**no.**	**p-value**
**Demographics**			
Children (<15 years old) (n=69)	20	49	**0.008**
Adults (≥15 years old) (n=154)	74	80	
Male (n=134)	59	75	0.486
Female (n=89)	35	54	
Recruited from Cochabamba (n=122)	63	59	**0.002**
Recruited from Santa Cruz (n=101)	31	70	
**History**			
HIV-positive (n=48)	26	22	0.057
HIV-negative (n=175)	68	107	
Hospital admission >48 hours (n=87)	35	52	0.641
**Aetiologies**			
Confirmed infectious aetiology (n=110)	52	58	0.081
Bacteria (n=41)	14	27	0.250
Mycobacteria (n=27)	15	12	0.132
Viruses (n=14)	7	7	0.539

Fungi (n=13)	6	7	0.763
Parasites (n=7)	3	4	0.969
Co-infection (n=8)	7	1	**0.010**
Probable infectious aetiology (n=22)	10	12	0.741
Possible infectious aetiology (n=4)	2	2	0.748
Non-infectious aetiology (n=48)	18	30	0.699
Unknown aetiology (n=39)	12	27	0.113
Respiratory infection (n=28)	8/66	20/102	0.203
**Signs and symptoms**			
Fever >37.5°C (n=193)	82	111	0.797
Neck stiffness or bulging fontanelle (n=99)	42	57	0.941
Glasgow coma scale <15 (n=138)	61/84	77/116	0.346
Glasgow coma scale <12 (n=74)	39/84	35/116	**0.020**
Seizures (n=93)	37	56	0.545
Headache (n=132)	53/91	79/119	0.226
Nausea and/or vomiting (n=135)	48	87	**0.013**
Hypoxemia <90% (n=96)	46/93	50/124	0.179
Tachypnea >20 breaths per minute (n=120)	51/93	69/127	0.940
**Clinical syndromes**			
Meningitis (n=47)	15	32	0.109
Encephalitis (n=36)	15	21	0.948
Meningoencephalitis (n=98)	46	52	0.199
**Blood analysis**			
Anaemia <12g/dL (n=123)	50	73	0.614
Leukocytosis >10,000 cell/mm^3 (n=107)	45	62	0.978
Neutrophilia >65% (n=180)	76	104	0.965
Band cells >3 cell/mm^3 (n=32)	19	13	**0.03**
Lymphocytosis >40% (n=15)	3	12	0.072
Thrombocytopenia <150,000 cell/mm^3 (n=40)	30	10/126	**<0.001**
Hyperglycaemia ≥130 mg/dL (n=41)	21/78	20/114	0.119
Hyponatremia <145 mEq/L (n=76)	36/92	40/114	0.549
Hypokalemia <3.5 mEq/L (n=40)	23/90	17/106	0.072
Hypochloremia <95 mEq/L (n=25)	10/66	15/79	0.519
Urea >45 mg/dL (n=50)	32/89	18/118	**<0.001**
Creatinine >1.3 mg/dL (n=31)	16/90	15/121	0.275
Hypoalbuminemia <3.5 g/dL (n=37)	18/35	19/50	0.219
C-reactive protein positive (n=38)	15/38	23/57	0.932
Aspartate transaminase >35 UI/L (n=79)	41/78	38/89	0.202
Alanine transaminase >41 UI/L (n=56)	30/78	26/90	0.189
Hyperbilirubinemia total >1.1 mg/dL (n=36)	17/60	19/74	0.729
Hypoproteinemia <6.1 g/dL (n=22)	9/25	13/39	0.826
Hyperglobulinemia >3.0 g/dL (n=38)	14/26	24/38	0.456
Rel Albumin/Globulin <1.1 (n=38)	14/21	24/34	0.759
Alkaline phosphatase >129 UI/L (n=44)	21/38	23/37	0.544
Gamma-glutamyltransferase >55 UI/L (n=10)	7/12	3/9	0.256
**Cerebrospinal fluid (CSF) analysis**			

Xanthochromia appearance (n=46)	27	19	**0.010**
Opalescent or turbid appearance (n=37)	16	21	0.883
Clear appearance (n=121)	42	79	**0.014**
Abnormal CSF (n=154)	70	84	0.135
Hypoglycorrhachia <40 mg/dL (n=130)	61	69	0.088
Elevated proteins >50 mg/dL (n=116)	46/91	70/128	0.545
Elevated leucocyte count >10 cell/mm^3 (n=113)	52	61	0.236
**Neuroimaging**			
No neuroimaging (n=73)	33	40	0.520
Normal (n=43)	13/61	30/88	0.090
Cerebral edema (n=36)	15/61	21/88	0.918
Hydrocephalus (n=17)	12/61	5/88	**0.008**
Brain abscess, space occupying lesions or ring-enhancing lesions (n=18)	9/61	9/88	0.400

Supplementary Table S11. Univariate analysis of risk factors associated with death in patients with suspected central nervous system infection. Bolivia, 2017-2018

Variables (p<0.05 in univariate)	Relative risk	Odds ratio	95% Confidence interval	p value
Adults (≥15 years old)	1.67	2.29	1.24-4.2	0.168
Recruited from Cochabamba	1.7	2.45	1.41-4.26	0.679
Glasgow coma scale <12	1.54	2.01	1.11-3.59	0.062
Thrombocytopenia <150,000 cell/mm3	**4.02**	**5.44**	**2.49-11.83**	**0.026**
Urea >45 mg/dL	2.36	3.12	1.60-6.05	0.794
Xanthochromia appearance	1.8	2.33	1.20-4.51	0.178
Co-infection	9.61	10.3	1.24-85.19	0.087
Hydrocephalus	**1.9**	**4.07**	**1.35-12.23**	**0.020**

Supplementary Table S12. Multivariate logistic regression analysis of significant risk factors associated with death in patients with suspected central nervous system infection. Bolivia, 2017-2018

	Sequelae n=66 no.	Full recovery n=63 no.	p-value
**Demographics**			
Children (<15 years old) (n=49)	20	29	0.066
Adults (≥15 years old) (n=80)	46	34	
Male (n=75)	39	36	0.822
Female (n=54)	27	27	
Recruited from Cochabamba (n=59)	32	27	0.521
Recruited from Santa Cruz (n=70)	34	36	
**History**			
HIV-positive (n=22)	17	5	**0.007**
HIV-negative (n=107)	49	58	
Hospital admission >48 hours (n=69)	34	35	0.568
**Aetiologies**			
Confirmed infectious aetiology (n=58)	38	20	**0.003**
Bacteria (n=27)	12	15	0.432
Mycobacteria (n=12)	11	1	**0.003**
Viruses (n=7)	3	4	0.651
Fungi (n=7)	7	0	**0.013**
Parasites (n=4)	4	0	0.119
Co-infection (n=4)	4	0	0.119
Probable infectious aetiology (n=12)	7	5	0.602
Possible infectious aetiology (n=2)	0	2	0.145
Non-infectious aetiology (n=30)	16	14	0.786
Unknown aetiology (n=27)	5	22	**<0.001**
Respiratory infection (n=20)	7/51	13/51	0.134
**Signs and symptoms**			
Fever >37.5°C (n=111)	56	55	0.687
Neck stiffness or bulging fontanelle (n=57)	31	26	0.514
Glasgow coma scale <15 (n=39)	18/61	21/55	0.323
Glasgow coma scale <12 (n=35)	23/61	12/55	0.063
Seizures (n=56)	24	32	0.098
Headache (n=79)	40/63	39/56	0.478
Nausea and/or vomiting (n=90)	42	48	0.135
Hypoxemia <90% (n=50)	27/63	23/61	0.559
Tachypnea >20 breaths per minute (n=69)	31/64	38/63	0.179
**Clinical syndromes**			
Meningitis (n=32)	16	16	0.879
Encephalitis (n=21)	8	13	0.190
Meningoencephalitis (n=52)	28	24	0.616
**Blood analysis**			
Anaemia <12g/dL (n=73)	41	32	0.194
Leukocytosis >10,000 cell/mm^3 (n=62)	28	34	0.189
Neutrophilia >65% (n=104)	56	48	0.213
Band cells >3 cell/mm^3 (n=13)	8	5	0.429

Lymphocytosis >40% (n=9)	6	3	0.334
Thrombocytopenia <150,000 cell/mm^3 (n=10)	7/65	3/61	0.225
Hyperglycaemia ≥130 mg/dL (n=20)	8/60	12/54	0.212
Hyponatremia <145 mEq/L (n=40)	24/59	16/55	0.195
Hypokalemia <3.5 mEq/L (n=17)	11/58	6/48	0.366
Hypochloremia <95 mEq/L (n=15)	10/45	5/34	0.399
Urea >45 mg/dL (n=18)	11/65	7/53	0.576
Creatinine >1.3 mg/dL (n=15)	10/65	5/56	0.282
Hypoalbuminemia <3.5 g/dL (n=19)	14/31	5/19	0.183
C-reactive protein positive (n=23)	9/25	14/32	0.554
Aspartate transaminase >35 UI/L (n=38)	26/53	12/36	0.141
Alanine transaminase >41 UI/L (n=26)	15/54	11/36	0.775
Hyperbilirubinemia total >1.1 mg/dL (n=19)	14/46	5/28	0.229
Hypoproteinemia <6.1 g/dL (n=13)	7/24	6/15	0.485
Hyperglobulinemia >3.0 g/dL (n=24)	15/24	9/14	0.912
**Cerebrospinal fluid (CSF) analysis**			
Xanthochromia appearance (n=19)	14	5	**0.033**
Opalescent or turbid appearance (n=21)	10	11	0.722
Clear appearance (n=77)	33	44	**0.021**
Abnormal CSF (n=84)	51	33	**0.003**
Hypoglycorrhachia <40 mg/dL (n=32)	41	28	**0.044**
Elevated proteins >50 mg/dL (n=70)	41/65	29	0.053
Elevated leucocyte count >10 cell/mm^3 (n=61)	34	27	0.325
**Neuroimaging**			
No neuroimaging (n=41)	23	18	0.408
Normal (n=30)	14/43	16/45	0.589
Cerebral edema (n=21)	9/43	12/45	0.527
Hydrocephalus (n=5)	4/43	1/45	0.155
Brain abscess, space occupying lesions or ring-enhancing lesions (n=9)	6/43	3/45	0.259

Supplementary Table S13. Univariate analysis of risk factors associated with sequelae in survivors with suspected central nervous system infection. Bolivia, 2017-2018

Variables (p<0.05 in univariate)	Relative risk	Odds ratio	95% Confidence interval	p value
HIV-positive	**1.69**	**4.0**	**1.3-11.7**	**0.030**
Confirmed infectious aetiology	1.66	2.9	1.4-6.0	0.879
Mycobacterial infection	**1.95**	**12.4**	**1.5-99.1**	**0.009**
Fungal infection	2.07	16.0	0.9-286.5	0.036
Xanthochromia appearance	1.56	3.12	1.05-9.26	0.318
Abnormal CSF	1.82	3.09	1.44-6.60	0.094
Hypoglycorrhachia <40 mg/dl	1.43	2.05	1.01-4.14	0.094

Supplementary Table 14. Multivariate logistic regression analysis of significant risk factors associated with sequelae in survivors with suspected central nervous system infection. Bolivia, 2017-2018

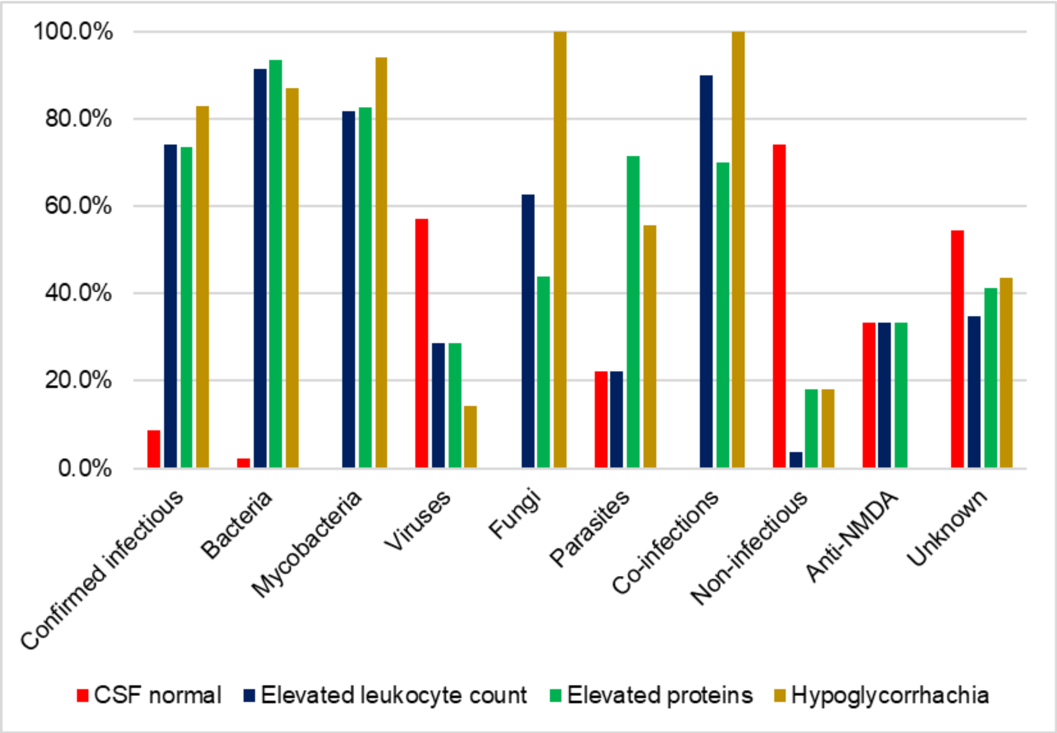

Supplementary Figure S1. Cerebrospinal fluid parameters in patients with suspected central nervous system infection. Bolivia, 2017-2018
